# Interventions for Infection and Inflammation-Induced Preterm Birth: a Preclinical Systematic Review

**DOI:** 10.1007/s43032-022-00934-x

**Published:** 2022-04-14

**Authors:** Faith A. Miller, Adalina Sacco, Anna L. David, Ashley K. Boyle

**Affiliations:** 1 Elizabeth Garrett Anderson Institute for Women’s Health, University College London, 86-96 Chenies Mews, London, WC1E 6HX UK; 2 National Institute for Health Research University College London Hospitals Biomedical Research Centre, London, UK

**Keywords:** Premature labour, Preterm birth, Lipopolysaccharide, Inflammation, Animal models, Therapeutics

## Abstract

Spontaneous preterm births (< 37 weeks gestation) are frequently associated with infection. Current treatment options are limited but new therapeutic interventions are being developed in animal models. In this PROSPERO-registered preclinical systematic review, we aimed to summarise promising interventions for infection/inflammation-induced preterm birth. Following PRISMA guidance, we searched PubMed, EMBASE, and Web of Science using the themes: “animal models”, “preterm birth”, “inflammation”, and “therapeutics”. We included original quantitative, peer-reviewed, and controlled studies applying prenatal interventions to prevent infection/inflammation-induced preterm birth in animal models. We employed two risk of bias tools. Of 4020 identified studies, 23 studies (24 interventions) met our inclusion criteria. All studies used mouse models. Preterm birth was most commonly induced by lipopolysaccharide (18 studies) or *Escherichia coli* (4 studies). Models varied according to infectious agent serotype, dose, and route of delivery. Gestational length was significantly prolonged in 20/24 interventions (83%) and markers of maternal inflammation were reduced in 20/23 interventions (87%). Interventions targeting interleukin-1, interleukin-6, and toll-like receptors show particular therapeutic potential. However, due to the heterogeneity of the methodology of the included studies, meta-analysis was impossible. All studies were assigned an unclear risk of bias using the SYRCLE risk of bias tool. Interventions targeting inflammation demonstrate therapeutic potential for the prevention of preterm birth. However, better standardisation of preterm birth models, including the dose, serotype, timing of administration and pathogenicity of infectious agent, and outcome reporting is urgently required to improve the reproducibility of preclinical studies, allow meaningful comparison of intervention efficacy, and aid clinical translation.

**Supplementary Information:**

The online version contains supplementary material available at 10.1007/s43032-022-00934-x.

## Introduction

Approximately 11% of births worldwide occur prematurely, defined as before 37 weeks of gestation [1]. A large proportion of preterm births, between 40 and 80%, are associated with infection and inflammation within the reproductive tract and gestational tissues, including the uterus, cervix, placenta, decidua, and fetal membranes, as well as the amniotic fluid [2–4]. Inflammation is an integral part of labour, whether term or preterm, as there is an influx of immune cells and an increase in pro-inflammatory mediator production, which initiates the parturition process [5–7]. Immune cells, predominantly neutrophils, monocytes, and macrophages, infiltrate reproductive tissues, where they may enhance myometrial contraction and contribute to cervical remodelling [8, 9]. The profiles of cytokines, particularly interleukin (IL)-1α, IL-1β, IL-6, IL-8, IL-10, and tumour necrosis factor-alpha (TNFα), are altered in cases of preterm birth (PTB) when inflammation can take on a pathological role [10–12].

While all infants born prematurely have an increased risk of neonatal morbidity and mortality, those born following intrauterine infection demonstrate poorer neurological outcomes than those without exposure to infection [13, 14]. Animal models have supported this causal link, providing evidence that inducing maternal inflammation even without triggering PTB can cause significant brain injury in offspring [15]. Current therapies, such as progesterone and cervical cerclage, as recommended by National Institute for Health and Care Excellence (NICE), are relatively ineffective at prolonging gestation and improving neonatal outcomes [14]. Furthermore, there are no treatments to repair brain injury in premature infants after birth. There is, therefore, an urgent clinical need to identify, prevent, and treat pathological inflammation in the gestational tissues, in order to protect the developing fetus from potential inflammation-induced injury, prolong gestation, and improve the long term health of the offspring [16].

Several animal models have been developed to investigate the underlying mechanisms of PTB, including many that induce parturition through exposure to inflammation or infection [16–27], and reviews have compared these preclinical models to human PTB [28–32]. Many therapeutics have since been tested in these preclinical models to determine their potential for clinical translation but, as yet, these data have not been consolidated.

The aim of this study was to perform a systematic review of published literature on interventions for infection and inflammation-induced PTB in preclinical animal models, to provide a summary of which therapies hold the potential to prevent infection/inflammation-induced PTB in humans, and to advise on the direction for future research in this field.

## Methods

The study protocol was registered on PROSPERO on 18/05/2020 (registration number: CRD42020182763) [33] and the review was undertaken in accordance with the Preferred Reporting Items for Systematic Reviews and Meta-Analyses (PRISMA) statement [34].

### Eligibility Criteria

The PICOS framework was used to outline eligibility criteria [35]. Exclusion criteria are summarised in Table 1.

**Table 1:** List of exclusion criteria applied to publications during screening and review

1	Non-original research articles, systematic reviews, review articles, abstracts, any non-peer reviewed literature
2	*In vitro* studies
3	Humans
4	Not a preterm birth model, animals with non-infection/inflammation-induced preterm birth (including Mifepristone (RU486), prostaglandins, alcohol, environmental agents) or preterm birth induced by genetic manipulation
5	No intervention or postnatal intervention
6	No suitable control for model
7	No suitable control for intervention
8	Gestation length not assessed
9	Maternal inflammation not assessed

#### Participants

All species of animal models of infection/inflammation-induced PTB.

#### Intervention

Prenatal interventions to prevent PTB. The study must have induced PTB using an infectious or pro-inflammatory agent, rather than other non-infectious/inflammatory mechanisms such as Mifepristone (RU486), prostaglandins, alcohol, and environmental agents.

#### Comparison

**Fig. 1:**
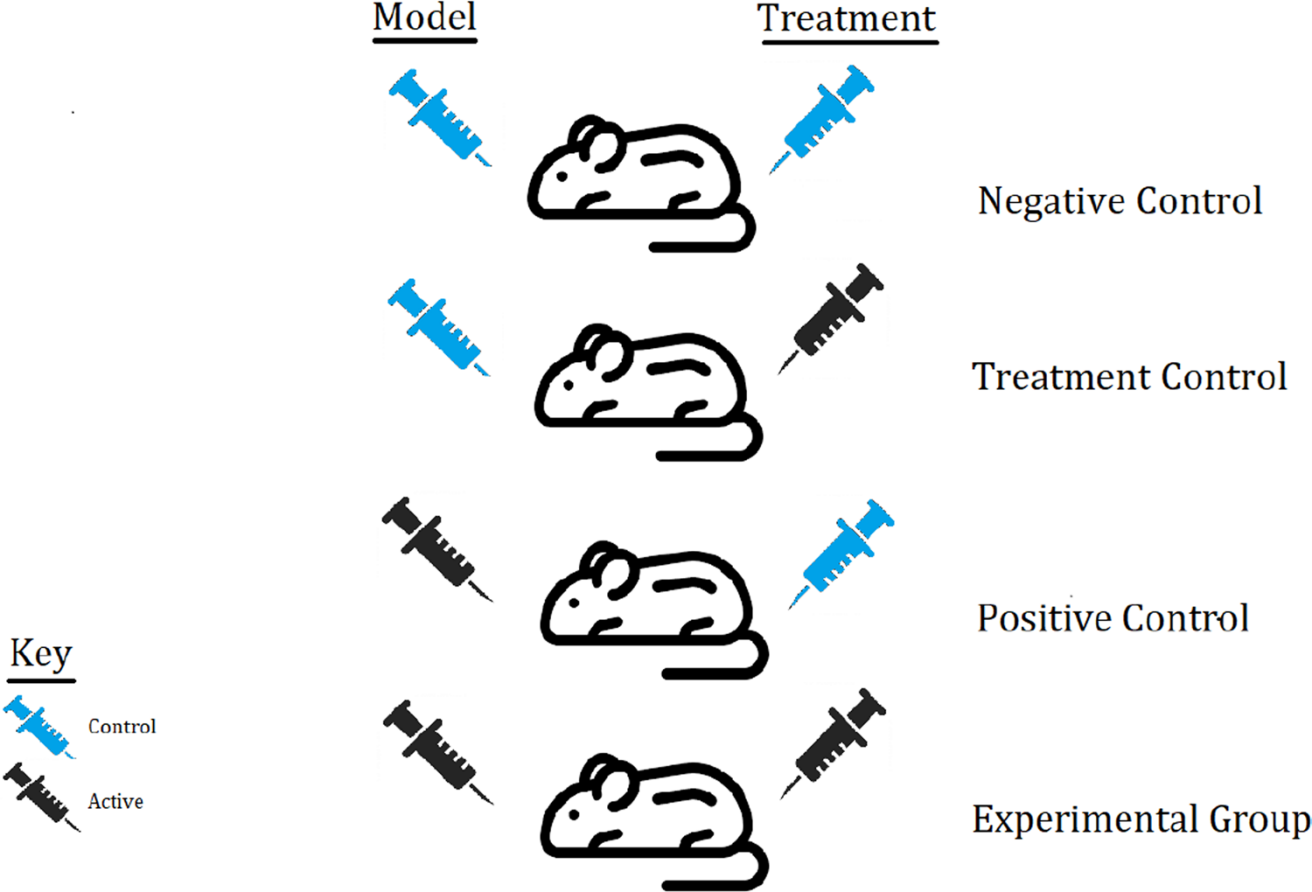
The control and experimental groups required for studies to be included in this systematic review. A requirement for inclusion was the incorporation of a negative control (with a vehicle control for the PTB model + a vehicle control for the intervention/treatment), treatment control (with a vehicle control for the PTB model + the active intervention/treatment), positive control (with the active PTB model + a vehicle control for the intervention/treatment), and experimental group (with the active PTB model + the active intervention/treatment)

The intervention was compared to an appropriate vehicle control. Due to the invasive nature of inducing PTB and delivering interventions, appropriate vehicle controls delivered in the same manner for both preterm model and intervention were a strict requirement. Figure 1 summarises the control groups that were required for inclusion; studies required a negative control (with a vehicle control for the PTB model and a vehicle control for the intervention/treatment), a treatment control (with a vehicle control for the PTB model and the active intervention/treatment), a positive control (with the active PTB model and a vehicle control for the intervention/treatment), and an experimental group (with the active PTB model and the active intervention/treatment).

#### Outcome

Required outcomes included the impact of the intervention on gestational length and maternal inflammation. Neonatal and pup survival and other fetal and maternal outcomes were included as additional outcomes but were not a requirement for study inclusion.

#### Study Type

Original quantitative, peer-reviewed, and controlled studies.

### Search Strategy

MEDLINE, EMBASE, and Web of Science databases were searched with no date or language restrictions. Searches were undertaken on 19/06/2020 and repeated on 20/01/2021. Search terms included a combination of free text and Medical Subject Headings (MeSH) terms, or equivalent, under the themes of “Animal Models”, “Preterm Birth”, “Inflammation”, and “Therapeutics”. Reference lists were also hand searched for further publications. All searches were undertaken by one researcher (FM). The complete search strategies are presented in Supplementary File 1.

### Study Selection

Two reviewers (FM and AB) independently screened all identified articles for eligibility using Covidence software (Melbourne, Australia) [36]. Initially, titles and abstracts were screened for eligibility according to the exclusion criteria in Table 1. The full texts of the remaining studies were then independently reviewed by FM and AB using the same criteria. Disagreements were resolved by discussion.

### Data Extraction

A list of fields to be extracted from each paper was established to determine the effect of the intervention on (i) gestational length, (ii) maternal inflammation, (iii) pup survival, and iv) other maternal, fetal, and offspring health outcomes. Data were also collected on publication details, PTB model, and study design. FM constructed an Excel spreadsheet for data entry, which FM and AB piloted through individually extracting data from three papers and comparing entries. This was used for data extraction. The two spreadsheets were then collated, and any discrepancies were checked against the full text. Data were extracted from text, tables, and/or graphs and were only extracted if the numerical results were clearly expressed by the authors; no assumptions were made.

### Assessment of Risk of Bias

To assess the quality of the selected studies and determine the presence of bias in their design, the SYRCLE risk of bias tool for animal intervention studies was applied. This tool is an adapted version of the Cochrane risk of bias tool, developed to capture the difference in methodology between animal intervention studies and clinical trials in humans [37]. A ‘high’ score indicates a high risk of bias, a ‘low’ score indicates a low risk of bias, and ‘unclear’ indicates an unknown risk of bias for each aspect of study design.

Several researchers have commented on the poor reporting of experimental design in animal studies, meaning a large proportion of bias outcomes are assigned an ‘unclear risk of bias’ [38, 39]. Menting et al. overcame this problem by adding three extra categories to assess bias more generally; whether researchers report any measure of randomisation, blinding, or power/sample size calculation [39]. This tool was also applied in this systematic review. These three outcomes were assigned either a ‘high’ or ‘low’ risk of bias. Studies were not excluded based on a high risk of bias. FM and AB independently assessed the risk of bias.

### Data Synthesis

Due to the heterogeneous nature of the PTB models and treatments, a meta-analysis of the data was not possible. Instead, individual outcomes were collated and analysed qualitatively according to the target of the intervention. Studies were grouped according to whether the intervention targeted inflammation/infection directly or indirectly.

## Results

### Study Selection

**Fig. 2:**
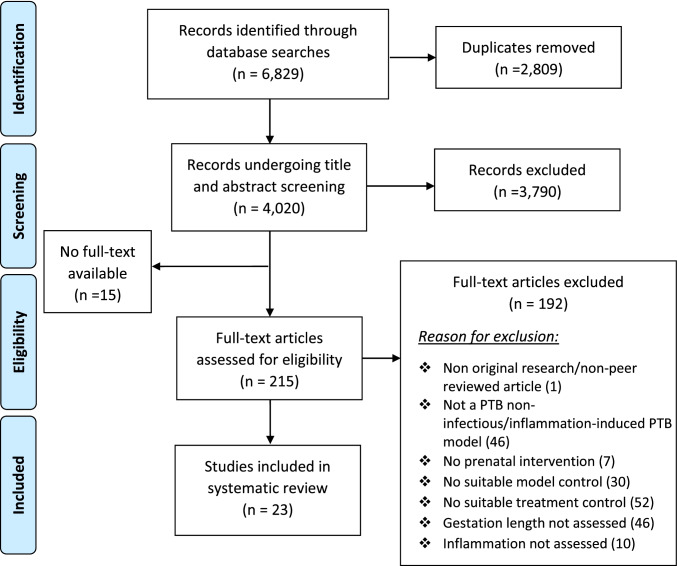
PRISMA flow diagram providing an overview of study selection for review. Adapted from Moher et al.^34^

Searches identified 6829 publications for review. A total of 2809 duplicates were identified and removed. Hand searching through reference lists of relevant papers identified no further literature. After title and abstract screening, 215 papers were selected for full-text review. Out of these, 23 studies were selected for inclusion. The PRISMA flow diagram, including reasons for exclusion, can be found in Fig. 2.

### Study Characteristics

Table 2 provides an overview of the characteristics of the preclinical PTB models used by the included studies. All studies used a mouse model [40–62]. Eighteen models (75%) induced PTB using LPS [40, 42–45, 47–51, 53–56, 58, 59, 61, 62], and four (17%) used *E. coli* [41, 46, 47, 60]. One study induced PTB with a monoclonal anti-CD3ε antibody [52] and another applied carbamyl-platelet activating factor (cPAF) [57]. In 13 models, the PTB agent was administered intraperitoneally (IP) [43, 44, 47, 48, 50, 52, 55–59, 61, 63]. Twelve models injected the PTB agent into the intrauterine (IU) space; 11 following a mini-laparotomy and one by ultrasound-guided injection [40–42, 45–47, 49, 51, 53, 54, 57, 60]. Sample sizes varied between 3 and 72 dams per group and the PTB agent was administered between gestation days (GD) 14 and 17.

**Table 2:** Study characteristics and method for inducing preterm birth grouped according to the agent used to induce PTB. All included studies were performed in the pregnant mouse

Paper	Mouse strain	# Dams/group	Agent used	Serotype	Dosage	Preparation	Administration route	Plug day designation	GA at PTB induction (days)
Agrawal et al., 2018 [60]	CD-1	3–15	*E.coli*	ATCC no. 12014	1–3 × 10^3 *E. coli* organisms	In 20 μl saline	IU injection	Unknown	14.5
Peltier et al., 2013 [41]	CD-1	10–21	*E.coli*	Unknown	10^6 CFU/100 μl	In 100 μl PBS	IU injection	Unknown	14
Filipovich et al., 2015 [46]	CD-1	4–10	*E.coli*	Unknown	2 × 10^7 or 6 × 10^7 CFU	In 100 μl PBS	IU injection	GD0.5	14.5 + 5 h
Chin et al., 2016 [47]	C57BL/6	10–20	*E. coli*	O55:K59(B5):H	5 × 10^10 CFU/100 μl	In 100 μl PBS	IU injection	GD0.5	16.5
			LPS	Unknown	0.5 μg	In 200 μl PBS	IP injection		
Lei et al., 2017 [49]	CD-1	4–72	LPS	O55:B5	25 μg	In 100 μl PBS	IU injection	Unknown	17
Shynlova et al., 2014 [44]	CD-1	6–11	LPS	055:B5	50 μg	In 100 μl saline	IP injection	GD0.5	15
Yang et al., 2014 [45]	CD-1	9–17	LPS	055:B5	125 μg	In 100 μl saline	IU injection	GD1	15
Toda et al., 2016 [59]	C3H/HeN	6–22	LPS	055:B05	2 × 50 μg/kg	In 200 μl saline	IP injection	GD0.5	15.5
Schander et al., 2020 [56]	BALB/c	16	LPS	05:B55	2 doses: 139 & 390 μg/kg	In saline	IP injection	GD0	15
Boyle et al., 2019 [53]	C56BL/6 J	10–25	LPS	0111:B4	1 μg	In 25 μl PBS	IU injection	GD1	17
Herbert et al., 2019 [54]	CD-1	5–14	LPS	0111:B4	10 μg	In 25 μl PBS	IU injection	GD0	16
Sykes et al., 2013 [42]	CD-1	4	LPS	0111:B4	20 μg	In 25 μl PBS	IU injection	GD0	16
Rinaldi et al., 2015 [51]	CD-1	9–12	LPS	0111:B4	20 μg	In 25 μl PBS	IU injection	GD1	17
Zhang et al., 2020 [62]	BALB/c	10–14	LPS	0111:B4	2 × 50 μg/kg	In saline	IP injection	GD0	15
Liu et al., 2016 [48]	BALB/c	6–12	LPS	0111:B4	400 μg/kg	In 100 μl saline	IP injection	GD0.5	14.5
Schmitz et al., 2007 [40]	CD-1	11–13	LPS	O127:B8	10 μg	In 100 μl PBS	IU injection	Unknown	15
Chen et al., 2012 [61]	CD-1	11	LPS	0127:B8	1–3 × 75 μg/kg	In saline	IP injection	GD0	15–17
Fu et al., 2019 [61]	CD-1	15–20	LPS	0127:B8	200 μg/kg	In saline	IP injection	GD0	15
Nadeau-Vallée et al., 2017 [55]	C57BL/6	10	LPS	Unknown	0.5 μg	In 200 μl PBS	IP injection	GD0.5	16.5
Madaan et al., 2017 [50]	CD-1	4–22	LPS	Unknown	10 μg	In 100 μl saline	IP injection	Unknown	16
Domínguez Rubio et al., 2014 [43]	BALB/c	10	LPS	Unknown	2 doses: 10 μg (260 μg/kg) & 20 μg (520 μg/kg)	In 100 μl saline	IP injection	GD0	15
Arenas-Hernandez et al., 2019 [52]	C57BL/6 J	5–10	Anti-CD3ε Ab	Unknown	10 μg	In 200 μl PBS	IP injection	GD0.5	16.5
Wahid et al., 2020 [57]	BALB/c	10–19	cPAF	Unknown	IP: 2 μg IU: 35 μg	In 100 μl PBS	IU or IP injection	GD0.5	16.5

*Ab*, *antibody*; *cPAF*, *carbamyl-platelet activating factor*; *CFU*, *colony-forming unit*; *GA*, *gestational age*; *GD*, *gestation day*; *IP*, *intraperitoneal*; *IU*, *intrauterine*; *LPS*, *lipopolysaccharide*; *PBS*, *phosphate-buffered saline*; *PTB*, *preterm birth*

Table 3 describes the six studies in which the intervention directly targeted infection/inflammation. Three interventions targeted the production or signalling of cytokines [44, 55, 59], two targeted leukocytes [46, 51], and one targeted the reproductive tract microbiome [45]. The remaining 17 studies, shown in Table 4, affect the inflammatory parturition pathway indirectly through adaptation of maternal physiology more generally. One study applied surfactant protein (SP)-A, a glycoprotein that affects toll-like receptor (TLR) signalling [60]. Two studies applied opioid receptors, which also target TLR signalling [47, 57]. Two interventions targeted prostaglandin production [40, 42]. Further studies applied recombinant erythropoietin (EPO) [62], 3,5-dihydroxybenzoic acid (3,5-DHBA), a GPR81 agonist [50], dendrimer-N-acetyl cysteine conjugate (DNAC) [49], and simvastatin [53]. One study housed animals in an enriched environment, in which cages had interactive objects such as tunnels, balls, nesting material, running wheels, and more animals per cage [56]. Two studies applied progesterone [52, 54], one with the cyclic-AMP-phosphodiesterase inhibitor aminophylline [54]. Further studies applied, melatonin [43], vitamin D [61], zinc [58], hydrogen sulphide [48], and carbon monoxide [41]. Comprehensive data extraction tables can be found in Supplementary File 2.

**Table 3:** Summary of the studies in which interventions directly targeted inflammation, and their effect on gestational length, neonatal survival, pup survival, and maternal inflammation

Paper	PTB agent	Therapeutic agent used	Function	Inflammatory target	Significant effect on* (↑/↓)				Sig. effect on inflammation* (↑/↓)
					Gestation length		Neonatal survival	Pup survival	
Toda et al., 2016 [59]	LPS	IMD-0560	IκB kinase β inhibitor	IL-6 production	↑	*p* < 0.001	-	-	Blood & uterus: ↓ IL-6, *Il-6*, *Kc*, *Mcp-1 & Mip-2*
	LPS	MR16-1	Anti IL-6R antibody	IL-6 production	↑	*p* < 0.05	-	-	-
Nadeau-Vallée et al., 2017 [55]	LPS	101.1	IL-1R antagonist	IL-1 signalling	↑	*p* < 0.05	↑	↑ @ 1 week	Uterus: ↓ *Il-1β*, *Il-6*, *Tnfα*, *Il-10* Decidua: ↓ *Il-1α*, *Il-1β*, *Il-6*, *Tnfα*, *Il-10*, *Il-12b* Placenta: ↓ *Il-1α*, *Il-1β*, *Il-6*, *Tnfα*, *Il-10*
Filipovich et al., 2015 [46]	*E. coli*	PMN antiserum	Antibody	PMN cell depletion	No	*p* > 0.05	No	-	Uterus & serum: ↓ total WBC & % neutrophils Uterus & placenta: ↓ myeloperoxidase & elastase in uterus
Rinaldi et al., 2015 [51]	LPS	15-epi-lipoxin A4	Arachidonic acid metabolite	Neutrophil metabolism	No	*p* > 0.05	↑	-	No
Shynlova et al., 2014 [44]	LPS	BSCI	Chemokine receptor inhibitor	Chemokine signalling and neutrophil activity	↑	*p* < 0.05	No	-	Plasma: ↓TNFα, IL-6, CSF2 Liver: ↓ *Il-1*β, *Il-12*, *Csf2*, *Ccl2*, *Ccl4*, *Cxcl1*, *Cxcl2* Myometrium: ↓ *Il-1β*, *Il-6*, *Il-12*, *Csf2*, *Ccl2*, *Ccl4*, *Cxcl1*, *Cxcl2*, neutrophil accumulation Decidua: ↓ *Il-1*β, *Il-12*, *Csf2*, *Ccl4* Placenta: ↓*Cxcl2*
Yang et al., 2014 [45]	LPS	*L. rhamnosus* GR-1	Probiotic	Microbiome	↑	*p* = 0.028	No	-	Plasma: ↓ IL-1β, IL-6 & IL-12p40, TNFα & CCL4/5 Myometrium: ↓IL-6, IL-12p70, IL-13, IL-17, TNFα & CSF2 Amniotic fluid: ↓ IL-6, TNFα & CCL3/4 Placenta: ↓ IL-6 & IL-12p70

*BSCI*, *broad-spectrum chemokine inhibitor*; *IL*, *interleukin*; *LPS*, *lipopolysaccharide*; *PMN*, *polymorphonuclear*; *R*, *receptor*; *TNF*; *tumour necrosis factor*; *WBC*, *white blood cells*; *WBC*. *Significant difference in *outcome** measure in experimental group compared with positive control.* “↑” increase; “↓” decrease; “*No*” *no statistically significant effect*; “*-*” *outcome was not assessed*

**Table 4:** Summary of the studies in which interventions indirectly targeted inflammation grouped according to their mechanism of action, and their effect on gestational length, neonatal survival, pup survival, and maternal inflammation

Paper	PTB agent	Therapeutic agent	Function	Inflammatory target	Significant effect on * (↑/↓)				Sig. effect on inflammation? *(↑/↓)
					Gestational length		Neonatal survival	Pup survival	
Agrawal et al., 2018 [60]	*E. coli*	SP-A	Surface glycoprotein	TLR signalling	↑	*p* < 0.0001	↑	-	Uterus: ↓ TNF, IL-6, IFN-γ, MIP-1β Reverse polarisation from M1 to M2 phenotype
Chin et al., 2016 [47]	LPS OR *E. coli*	Naloxone	Opioid receptor antagonist	TLR signalling	↑	*p* < 0.05	↑	↑ @ 3 weeks	Fetal membrane: ↓ *Il-1β*, *Il-6*, *Tnfα*, *Il-10* Uterus: ↓ *Il-1α*, *Tnfα* Myometrium: ↓ *Tnfα*
Wahid et al., 2020 [57]	cPAF	Naltrexone	Opioid receptor antagonist	TLR signalling	↑	*p* < 0.05	↑	↑ @ 3 weeks	Uterus: ↓ *Il-1β*, *Il-6*, *Il-10*, *Ptgs2* Myometrium: ↓ *Il-6*, *Il-12b*, *Il-10* Placenta: ↓ *Il-1β*, *Il-6*
Schmitz et al., 2007 [40]	LPS	Rolipram	PDE4 inhibitor	Prostaglandin production	↑	*p* < 0.05	↑	-	Amniotic fluid: ↓ TNFα, IL-1β & IL-6 Decidua: ↓ uNK cell recruitment & NFκB nuclear translocation
Sykes et al., 2013 [42]	LPS	Pyl A	CRTH2 agonist	Prostaglandin production	↓	*p* < 0.01	↑	-	Myometrium: ↑ IL-12, IL-1β, KC-GRO, *Ifn-γ*, *Tnfα*
Zhang et al., 2020 [62]	LPS	EPO	Hormone	Jak2/STAT3 activation	↑	*p* < 0.0001	↑	-	Serum & amniotic fluid: ↓ IL-1β, IL-6 & TNF Placenta: ↓ leukocyte infiltration Placenta: PD-L1 ↑ Uterus: ↓ NFκB activation (*p65*) & iNOS
Madaan et al., 2017 [50]	LPS	3,5-DHBA	GPR81 agonist	Lactate signalling	↑	*p* < 0.001	↑	-	3,5-DHBA: Uterus: ↓ *Il-6*, *Ccl2*
Lei et al., 2017 [49]	LPS	DNAC	Antioxidant	Macrophage cytokine production	↑	*p* = 0.004	-	-	Placenta: ↑ *Il-10* Placenta: ↓ infiltration of CD3+ T-cells & CD8+ T-cells
Boyle et al., 2019 [53]	LPS	Simvastatin	HMG-CoA reductase inhibitors	Metabolism	↑	*p* < 0.05	No	-	Serum: ↓ IL-6 Uterus: ↓ *Il-6*, *IL-10*, *Cxcl1*, *Ccl2*
Schander et al., 2020 [56]	LPS	EE	Stress reduction	Stress induced inflammation	↑	*p* < 0.05	No	-	Uterus: ↓ TLR4, CD14 & neutrophil infiltration
Arenas-Hernandez et al., 2019 [52]	Anti-CD3ε Ab	Progesterone	Hormone	Unclear	↑	*p* < 0.001	↑	-	Decidua: ↓ *Ccl22*, *Icam1*, *Ctka4*, *Nod1*, *Ccl5* Cervix: ↓ *Il-6*, *Il-12b*, *Il-1α*, *Pycard*, *Il-4* Myometrium: ↓ *Il-33*
Herbert et al., 2019 [54]	LPS	Am & P4 combined	P4: hormone Am: cAMP-PDE inhibitor	Unclear	↑	*p* < 0.001	No	-	Uterus: ↓ IL-6, CCL1, IL-11, IRAK3, *Il-6*
Domínguez Rubio et al., 2014 [43]	LPS	Melatonin	Hormone	Unclear	No	*p* > 0.05	No	-	Uterus: ↓ TNFα, NOS activity, iNOS
Fu et al., 2019 [61]	LPS	Vitamin D	Secosteroid hormone	Unclear	↑	*p* < 0.05	↑	-	Placenta: ↓ p-IκBα, IκBα, NF-κB p65, NF-κB p50 & p65 + nucleus, *Tnfα*, *Il-1β*, *Mcp1*, *Mip2*
Chen et al., 2012 [61]	LPS	Zinc	Signalling molecule	Unclear	↑	*p* < 0.05	↑	-	Placenta & serum: ↓ IL-1β, IL-6, TNFα, IL-8, *Il-1β*, *Il-6*, *Tnfα*, *IL-8*, NFκB activation Placenta & serum: ↑ IL-4, IL-10, *Il-4*, *Il-10*
Liu et al., 2016 [48]	LPS	Hydrogen sulphide	Signalling molecule	Unclear	↑	*p* < 0.05	-	-	Uterus: ↓ leukocyte infiltration Serum: ↓ IL-6, TNFα, CCL2, CXCL15 Myometrium: ↓ *Il-1β*, *Il-6*, *Tnfα*, *Ccl2*, *Cxcl15* Placenta: ↓ ERK1/2, p65, *Il-6*, *Tnfα*
Peltier et al., 2013 [41]	*E. coli*	CO	Signalling molecule	Unclear	↑	*p* = 0.001	No	-	No

*Am*, *aminophylline*; *cAMP*, *cyclic adenosine monophosphate*; *CO*, *carbon monoxide*; *CRTH*, *chemoattractant receptor-homologous molecule*; *DHBA*, *dihydroxybenzoic acid*; *DNAC*, *N-acetyl cysteine*; *EE*, *enriched environment*; *EPO*, *erythropoietin*; *GPR*, *G protein-coupled receptor*; *HMG CoA*, *β-hydroxy β-methylglutaryl-CoA*; *IL*, *interleukin*; *LPS*, *lipopolysaccharide*; *NF*, *nuclear factor*; *P4*, *progesterone*; *PDE*, *phosphodiesterase*; *SP*, *surfactant protein*; *TLR*, *toll-like receptor*; *TNF*, *tumour necrosis factor*; *WBC*, white blood cells. *Significant difference in *outcome** measure in experimental group compared with positive control.* “↑” increase; “↓” decrease; “*No*” *no statistically significant effect*; “*-*” *outcome was not assessed*

### Gestational Length

As summarised in Table 3, five out of seven interventions (from six studies) that directly targeted inflammation significantly increased gestational length in the experimental group when compared with the positive control group (*p* < 0.05). The positive control group received an active PTB model and a vehicle control for the treatment (Fig. 1). The gestational length outcome was reported using various measures: either the proportion of dams delivering prematurely, time from PTB induction to delivery or gestational length, or a combination of these measures. Both methods of IL-6 inhibition significantly delayed birth (*p* < 0.05 or *p* < 0.001) [59], as did interference with IL-1 signalling using 101.1, the IL-1 receptor (IL-1R) antagonist (*p* < 0.05) [55]. Application of the broad-spectrum chemokine inhibitor (BSCI) [44] and promotion of *L. rhamnosus* dominance through the application of the probiotic GR-1 [45] significantly delayed labour when compared with the positive control (*p* < 0.05). Targeting leukocyte activity through depletion of polymorphonuclear (PMN) cells [46] or application of 15-epi-lipoxin A4, an arachidonic acid metabolite involved in neutrophil metabolism [51], had no significant effect on gestational length compared to the positive control groups (*p* > 0.05).

Fifteen of the 17 studies indirectly targeting inflammation found a significant increase in gestational length between the positive control and experimental group, as shown in Table 4. The surface glycoprotein SP-A [60], the opioid receptor antagonists naloxone [47] and naltrexone [57], and the phosphodiesterase type-4 (PDE4) inhibitor rolipram [40] all significantly increased gestational length, as did administration of EPO [62], 3,5-DHBA [50], DNAC [49], and simvastatin [53]. Housing mice in an enriched environment also significantly increased gestational length (*p* < 0.05) [56]. Progesterone, on its own [52] or with aminophylline [54], significantly increased gestational length, as did vitamin D [61], zinc [58], hydrogen sulphide [48], and carbon monoxide [41]. Conversely, application of Pyl A, a CRTH2 (chemoattractant receptor-homologous molecule expressed on TH2 cells) agonist, significantly reduced gestational length in the experimental group compared with the positive control group (*p* < 0.01) [42]. Melatonin did not exert an effect on gestational length (*p* > 0.05) [43].

### Maternal Inflammation

Researchers assessed inflammation in a variety of tissues and fluids, including plasma, liver, myometrium, decidua, placenta, and amniotic fluid, and assessed either the expression of messenger RNA (mRNA), protein levels, or the translocation of immune cells. Of the six studies directly targeting inflammation, five found that their intervention significantly reduced maternal inflammation in comparison to the positive control group (Table 3). Administration of 15-epi-lipoxin A4 did not exert any effect on the expression of proinflammatory markers in the PTB model [51], whereas IMD-0560 [59], 101.1 [55], PMN antiserum [46], BSCI [44], and *L. rhamonus* GR-1 [45] all exerted a significant reduction in maternal inflammation.

Of the 17 studies indirectly targeting inflammation, all but one (carbon monoxide [41]) exerted a significant effect on inflammation (Table 4).

### Neonatal Survival

Twenty studies reported on neonatal survival, describing either the proportion of live pups delivered, or proportion of pups alive *in utero* at a specified time after LPS/*E. coli* administration. Five of the six studies that directly targeted inflammation reported on the effect of the intervention on neonatal survival, with only 101.1 and 15-epi-lipoxin A4 having a significant effect (Table 3). While 15-epi-lipoxin A4 had no impact on gestational length, it did significantly improve neonatal survival in the experimental group compared with the positive control [51]. Neither the depletion of PMN cells [46], application of BSCI [44], nor administering *L. rhamnosus* GR-1 [45] significantly increased neonatal survival.

Of the 17 studies targeting inflammation indirectly, 15 reported on neonatal survival and ten reported a significant effect (Table 4). Administration of SP-A [60], naloxone [47], naltrexone [57], rolipram [40], Pyl A [42], EPO [62], 3,5-DHBA [50], progesterone [52], vitamin D [61], and zinc [58] significantly improved neonatal survival in the experimental group compared with the positive control. The combined administration of progesterone and aminophylline [54] had no significant effect on neonatal survival. Nor did simvastatin [53], housing mice in an enriched environment [56], melatonin [43], or carbon monoxide [41].

### Pup Survival

Three out of the 23 studies reported on pup survival, reporting survival between ages 1 and 3 weeks. Antagonism of IL-1R using 101.1 significantly improved pup survival at aged 1 week [55]. The two opioid receptor antagonists, naloxone [47] and naltrexone [57], also significantly improved pup survival at aged 3 weeks in the experimental group compared with the positive control. Additional fetal and maternal outcomes extracted from these studies can be found in Supplementary File 2.

### Risk of Bias

The risk of bias assessment is shown in Table 5. Eighty-five percent of outcome measures assessed using the SYRCLE risk of bias tool were assigned an unclear risk of bias. The outcome that was most frequently assigned a low risk of bias was ‘other problems that could result in high risk of bias’ (18 of 23), as determined using the authors ‘conflict of interest’ statement. The ‘groups [being] similar at baseline’ outcome was assigned a low risk of bias if the paper reported that the mouse strain and age/weight were kept consistent (in 12 out of 23 studies) [42, 45, 47, 48, 52, 53, 55–59, 61]. Three studies were assigned a ‘high’ risk of bias for selective outcome reporting, as authors did not report data on neonatal survival at birth [48, 57, 59], and one was assigned a ‘high’ risk of bias for reporting that caregivers were not blinded due to the nature of the animal’s housing [56].

**Table 5:** Risk of bias assessment

	Tool developed by Menting et al. [39]			SYRCLE risk of bias tool [37]									
Paper	Randomisation	Blinding	Power/sample size calculation	Was the allocation sequence adequately generated and applied?	Were the groups similar at baseline or adjusted for confounders in the analysis?	Was the allocation adequately concealed?	Were the animals randomly housed during the experiment?	Were the caregivers/investigators adequately blinded during the experiment?	Were animals selected at random during outcome assessment?	Was the outcome assessment blinded?	Were incomplete outcome data adequately addressed?	Are reports of the study free of selective outcome reporting?	Was the study apparently free of other problems that could result in high risk of bias? *i.e*., *Conflict of interest statement*
Agrawal et al., 2018 [60]	*High*	*High*	*High*	*?*	*?*	*?*	*?*	*?*	*?*	*?*	*?*	*?*	*?*
Arenas-Hernandez et al., 2019 [52]	*High*	*High*	*High*	*?*	*Low*	*?*	*?*	*?*	*?*	*?*	*?*	*?*	*Low*
Boyle et al., 2019 [53]	*Low*	*Low*	*Low*	*?*	*Low*	*?*	*?*	*?*	*?*	*?*	*?*	*?*	*Low*
Chen et al., 2012 [61]	*Low*	*High*	*High*	*?*	*Low*	*?*	*?*	*?*	*?*	*?*	*?*	*?*	*Low*
Chin et al., 2016 [47]	*High*	*High*	*High*	*?*	*Low*	*?*	*?*	*?*	*?*	*?*	*?*	*?*	*Low*
Domínguez Rubio et al., 2014 [43]	*High*	*High*	*High*	*?*	*?*	*?*	*?*	*?*	*?*	*?*	*?*	*?*	*Low*
Fu et al., 2019 [61]	*Low*	*High*	*High*	*?*	*Low*	*?*	*?*	*?*	*?*	*?*	*?*	*?*	*Low*
Herbert et al., 2019 [54]	*Low*	*High*	*Low*	*?*	*?*	*?*	*?*	*?*	*?*	*?*	*?*	*?*	*High*
Lei et al., 2017 [49]	*High*	*High*	*High*	*?*	*?*	*?*	*?*	*?*	*?*	*?*	*?*	*?*	*Low*
Liu et al., 2016 [48]	*High*	*High*	*High*	*?*	*Low*	*?*	*?*	*?*	*?*	*?*	*?*	*High*	*Low*
Madaan et al., 2017 [50]	*Low*	*High*	*High*	*?*	*?*	*?*	*?*	*?*	*?*	*?*	*?*	*?*	*Low*
Peltier et al., 2013 [41]	*High*	*High*	*High*	*?*	*?*	*?*	*?*	*?*	*?*	*?*	*?*	*?*	*High*
Schander et al., 2020 [56]	*Low*	*High*	*Low*	*?*	*Low*	*?*	*?*	*High*	*?*	*?*	*?*	*?*	*Low*
Schmitz et al., 2007 [40]	*High*	*High*	*High*	*?*	*?*	*?*	*?*	*?*	*?*	*?*	*?*	*?*	*Low*
Sykes et al., 2013 [42]	*High*	*High*	*High*	*?*	*Low*	*?*	*?*	*?*	*?*	*?*	*?*	*?*	*Low*
Wahid et al., 2020 [57]	*High*	*High*	*High*	*?*	*Low*	*?*	*?*	*?*	*?*	*?*	*?*	*High*	*Low*
Zhang et al., 2020 [62]	*High*	*High*	*High*	*?*	*?*	*?*	*?*	*?*	*?*	*?*	*?*	*?*	*Low*
Filipovich et al., 2015 [46]	*High*	*High*	*High*	*?*	*?*	*?*	*?*	*?*	*?*	*?*	*?*	*?*	*Low*
Nadeau-Vallée et al., 2017 [55]	*High*	*High*	*High*	*?*	*Low*	*?*	*?*	*?*	*?*	*?*	*?*	*?*	*High*
Rinaldi et al., 2015 [51]	*High*	*High*	*High*	*?*	*?*	*?*	*?*	*?*	*?*	*?*	*?*	*?*	*Low*
Shynlova et al., 2014 [44]	*Low*	*High*	*High*	*?*	*?*	*?*	*?*	*?*	*?*	*?*	*?*	*?*	*Low*
Toda et al., 2016 [59]	*High*	*High*	*High*	*?*	*Low*	*?*	*?*	*?*	*?*	*?*	*?*	*High*	*High*
Yang et al., 2014 [45]	*Low*	*High*	*High*	*?*	*Low*	*?*	*?*	*?*	*?*	*?*	*?*	*?*	*Low*

When applying the tool designed by Menting et al. [39], eight studies stated that they randomised mice into the model and intervention groups [44, 45, 50, 53, 54, 56, 58, 61], one described investigator blinding [53], and three stated that they had used a power or sample size calculation to determine their group sizes [53, 54, 56].

## Discussion

Animal models of infection/inflammation-induced PTB provide invaluable insight into the mechanisms involved in this common obstetric disorder. This systematic review established a thorough search strategy that identified 23 studies investigating prenatal interventions to prevent infection/inflammation-related PTB in mouse models. These studies consistently found that targeting inflammation within the reproductive tract can prevent preterm birth and improve neonatal outcomes in mice. Of the 24 interventions described in the 23 studies, nineteen found that their intervention significantly increased gestational length, and 12 out of 20 studies found their intervention significantly improved neonatal survival. It was difficult to draw firm conclusions regarding specific mechanisms due to the heterogeneity of the interventions and quality of the methodological reporting in the included studies.

### Research Design

A key finding of this review is that a large proportion of studies undergoing full-text review were excluded as they lacked all the necessary control groups; 82 studies were excluded for this reason. Eleven out of 23 studies included in our review involved a surgical procedure (i.e. mini-laparotomy), which causes neutrophil infiltration into the uterine tissues even in the sham control groups [64, 65]. This is clinically-relevant as non-obstetric abdominal surgery during the third trimester of pregnancy is associated with PTB in humans [66]. Therefore, one cannot draw firm conclusions regarding the effect of an intervention without a proper control to account for the creation of the PTB model itself. Furthermore, without a treatment only control group, one cannot be certain that the treatment alone does not cause detrimental effects. Therefore, for a study to be robust, it is essential that both the PTB model creation and treatment control groups are included.

### Species Diversity

A limitation of this review was that only studies using mouse models of PTB met our inclusion criteria. Mice are widely used in research due to their cost-effectiveness, short gestation period and life span for rapid data collection, and well-defined physiology and genetics [67, 68]. There is also extensive conservation in the immune response between mice and humans [69]. However, mice are small and, therefore, less resistant to surgical procedures. Their short gestation also prevents the study of chronic inflammation, which is particularly relevant for observing the effect of long-term exposure to infection/inflammation *in utero* [29]. Furthermore, there are vast differences in the physiology of labour in rodents and humans; systemic withdrawal of progesterone precedes labour in rodents, whereas it is understood that humans undergo a ‘functional progesterone withdrawal’ before labour [70, 71]. Of course, it is paramount that therapeutics are also tested in other large animal models such as non-human primates, for example. However, studies in large animals failed to meet the criteria of this review. Macaque studies were commonly excluded from review due to lacking the required control groups, likely due to the ethical and financial barriers to performing studies in non-human primates. Ovine models were commonly excluded as gestational length was our primary outcome and sheep do not tend to deliver preterm. Instead, ovine models are utilised to investigate fetal outcomes, for which they are a superior model to mice. Similarly, guinea pig studies were excluded as they focused on offspring outcomes rather than gestational length. Of all rodents, guinea pigs are the most similar to humans in terms of parturition and labour. They have a longer gestational period and, therefore, a greater proportion of brain development occurs *in utero*. They also have similar placental anatomy to humans, and they undergo a functional progesterone withdrawal in labour [72]. While significant differences in their physiology remain, a future review addressing interventions in these large animal models, with a stronger focus on fetal outcomes, could provide additional insight.

### Heterogeneity of Preterm Birth Models

We observed a high degree of heterogeneity in the PTB models. Each of the 23 studies induced PTB in a different manner, with variation in the mouse strain, PTB agent, serotype, dosage, and route and timing of administration. These factors all alter host response to the infectious agent. In studies administering LPS, doses ranged from 0.5 to 125 μg per mouse (with some studies calculating dose/kg which varies from 75 to 780 μg/kg; Table 3), with higher doses administered intraperitoneally (0.5–400 μg) compared to intrauterine (1–125 μg). There was also variation in the serotypes of LPS and *E. coli* administered. Different LPS serotypes induce different inflammatory pathways in the mother and pup brains, due to the way their structural variations interact with host TLRs [73]. Differences in the strain of mouse utilised also limits model comparison due to differences in inflammatory cell and cytokine response between mouse strains [74]. The timing of PTB induction varied from GD14-17 and plug day was designated either GD0, GD0.5, or GD1, with several studies not reporting on the plug day designation at all. There was also variation in the timing of administration of the preventative agent. This heterogeneity and ambiguity made it difficult to compare results and a meta-analysis was rendered impossible [19]. Furthermore, this heterogeneity also hinders the reproducibility of results. One argument is that this heterogeneity ensures treatments are effective in a variety of inflammatory models, reflecting the variation of inflammatory responses clinically. However, application of treatments in standardised models must preceed this. Clinical translation of therapeutic interventions administered during pregnancy requires a higher level of confidence in their effectiveness and safety, compared to those not administered in pregnancy, with many regulatory authorities requiring more than one pre-clinical study [75]. Standardised mouse models should use the same mouse strain and same dose, serotype, and method of administration of LPS or *E. coli*, in order to control for as many potential confounders as possible and increase the reproducibility of preclinical studies. Alternatively, guidelines could suggest a PTB morbidity rate to be achieved by models. However, it would be difficult to specify which rate would be most appropriate; models achieving 100% preterm birth commonly result in severe mobidity and even mortality of mother and pup. However, lower rates would require a larger number of mice to power studies sufficiently.

### Model Limitations

A limitation of the models included in this review is the route of administration. Clinically, PTB is induced following an infection ascending from the vagina via the reproductive tract [76]. However, the models in this review administered *E. coli* or LPS into the peritoneal cavity or uterus, inducing a systemic infection. Less invasive methods of inducing PTB which match the aetiology in humans would be clinically useful. Research groups have developed models of inducing PTB through intravaginal administration of *E. coli*, resulting in an ascending infection [77, 78]. However, administering LPS in the same manner has yielded inconsistent results [79–81]. Pavlidis et al. developed an ascending infection model of PTB using intravaginal administration of *Ureaplasma parvum*, which is commonly isolated from the reproductive tissues of women delivering preterm [82]. Furthermore, a modified approach to intrauterine LPS administration using ultrasound guidance, rather than mini-laparotomy, has been developed to reduce systemic inflammatory activation [81]. Further application of these more clinically translatable models is needed.

### Risk of Bias

The quality of reporting on methodological design was limited, with most outcomes in the SYRCLE risk of bias tool assigned an unclear risk of bias. A high risk of bias for selective outcome reporting was inferred if researchers did not report on neonatal survival, as we believe these data would have been available at the time of gestational length data collection and, therefore, deliberately withheld from the publication, possibly due to an unfavourable outcome. Results from the tool developed by Menting et al. were more informative [39]. Standards of reporting have improved since the incorporation of the ARRIVE (Animal Research: Reporting *In Vivo* Experiments) guidelines in 2010, which provide recommendations for reporting to improve quality of conduct [83, 84]. However, adherence to these guidelines is poor, contributing to the lack of reproducibility of experimental findings in animal research, delaying translation of these promising therapeutics into clinical use [38, 85]. While many journals state that adherence to the ARRIVE guidelines is mandatory, the verification process to ensure adherence could be improved. One form of bias we were unable to assess in our review is publication bias, in which negative results are withheld from publication [86]. This has most likely impacted our review to the high proportion of positive results from the included studies.

### Potential Therapeutic Targets

**Fig. 3:**
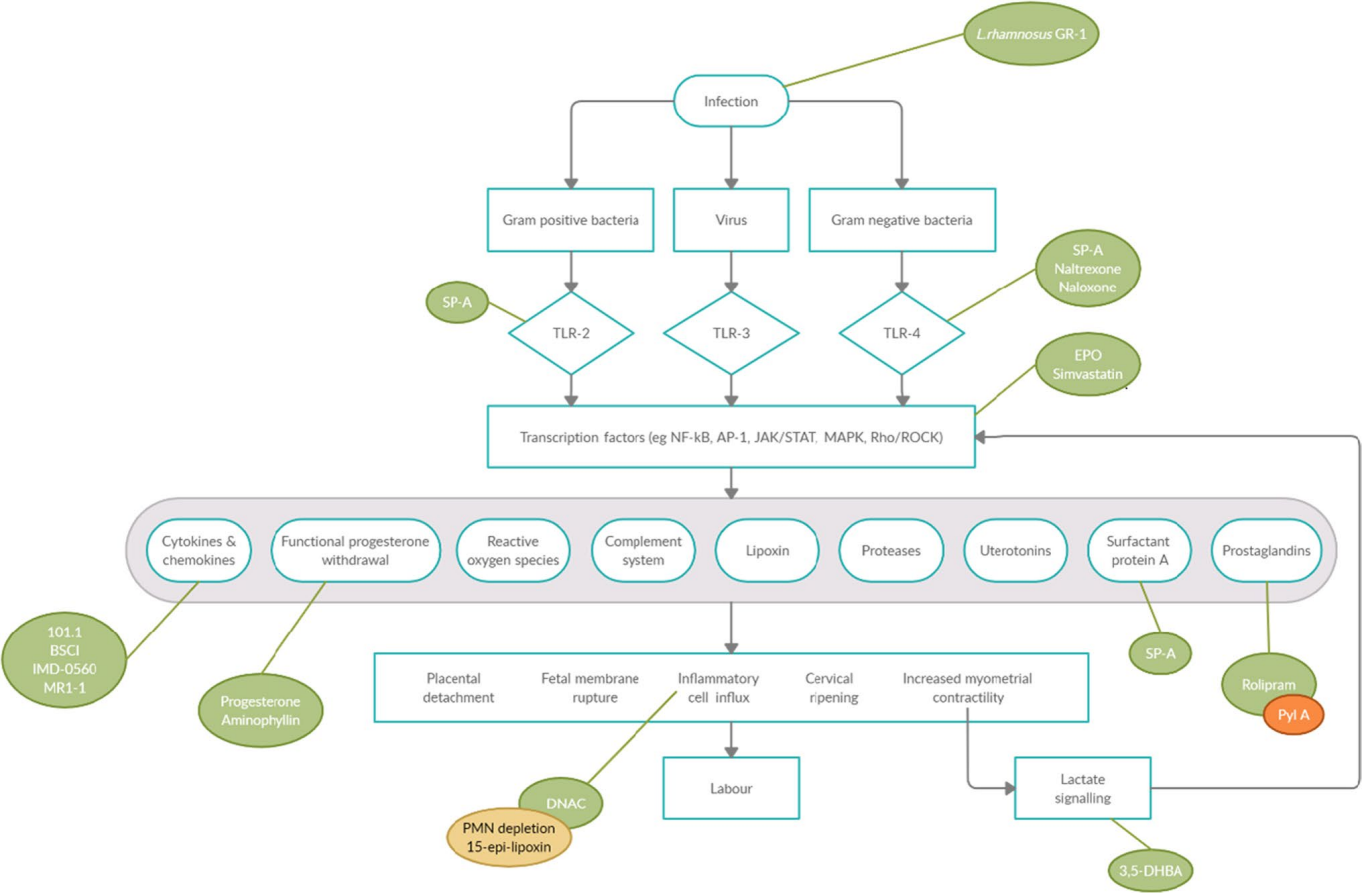
Summary of the known inflammatory targets of the interventions included in this review. Summary of pathways involved in infection/inflammation-induced PTB (adapted from Agrawal et al. [96]). Activation of toll-like receptors (TLRs) and other receptors by micro-organisms activates the proinflammatory immune cascade, controlled by transcription factors such as NFκB. This stimulates the activity of cytokines, prostaglandins, proteases, and enzymes, co-ordinating placental detachment, infiltration of inflammatory cells, cervical ripening, fetal membrane weakening, and uterine contractions, resulting in labour. The proposed mechanism of action of each intervention is included if known. Each intervention is colour coded according to its effect at delaying gestation in a PTB animal model; green, significantly prolongs gestation; yellow, no effect; red, significantly reduces gestation

The broad range of targets included in this review, as summarised in Fig. 3, demonstrates the complexity of the inflammatory mechanisms involved in PTB in mice. We have summarised the most promising targets from the included studies below.

Most interventions that increased gestational length were accompanied by a reduction in cytokine expression and activity. IL-6 in particular holds potential as a biomarker and tocolytic target [87]. Toda et al. found that suppression of IL-6 through inhibition of IκB kinase (IKK)-b using IMD-0560 prevented LPS-induced PTB [59]. A cohort study of Danish women taking sulfasalazine, an IKK inhibitor, for treatment of Crohn’s disease found that sulfasalazine is associated with an almost 50% reduction in PTB (compared with disease matched controls), suggesting it could hold promise as a preventative treatment for PTB [88]. However, sulfasalazine has significant cytotoxic effects which could be harmful and contribute to labour [89]. Furthermore, tocilizumab, an anti-IL-6 therapy, has been associated with an increased risk of PTB in humans. However, this has not been tested in a clinical trial [90]. IL-1 is another cytokine that has been indicated for its role in PTB and inducing the fetal inflammatory response [11]. Administration of 101.1, an IL-1R antagonist, was able to prolong gestation and protect the fetus from harmful exposure to IL-1, improving pup survival at 1 week [55]. The authors have subsequently shown that 101.1 reduces inflammatory retinopathy associated with PTB in mice, highlighting its potentially beneficial effect on neonatal outcomes [91].

Central to the activation and amplification of inflammation following microbial invasion is the activation of TLRs by microorganisms. All three studies targeting TLRs in this review found a significant increase in gestational length [47, 57, 60]. Naltrexone and naloxone, TLR4 antagonists, are already used clinically to counter the effects of opioids and treat alcohol dependence and are, therefore, known to be safe medications [92, 93]. However, they have both been found to cross the placenta and enter the fetal brain, where their effects are not entirely understood [94]. Neither study reported on pup neurological outcomes and, thus, further investigation is required [47, 57]. SP-A, which targets both TLR2 and TLR4, exerted a highly significant increase in gestational length, indicating that co-targeting of multiple TLR subtypes holds further promise in the treatment of PTB [60].

The involvement of prostaglandins in parturition has been well documented. However, the studies included in this review demonstrated conflicting results, with Sykes et al. finding that the CRTH2 agonist Pyl A augmented LPS-induced PTB rather than prolonging gestation as expected [40, 42]. Despite its effect on gestation length, Pyl A significantly improved neonatal survival, suggesting that different mechanisms are at play in each outcome [42, 51]. Furthermore, 15-epi-lipoxin A4, which had no significant effect on gestation length, also significantly improved neonatal survival. The authors suggest this is mediated through increased prostaglandin production, which may resolve the inflammatory environment surrounding the fetus and improve outcomes [51, 95]. Only three of the studies included in this review reported on long-term (between 1 and 3 weeks) health outcomes for the pups [47, 55, 57]. Further research on the health of these pups as they age would determine the longer-term consequences of these interventions.

### Strengths and Limitations of This Review

This review has several strengths. We applied strict inclusion criteria with regard to research design, to ensure only studies with reliable results were included. Furthermore, undertaking two risk of bias tools enabled thorough reporting on the bias of preclinical studies.

The main limitation of this review is that we were unable to meta-analyse the data due to the heterogeneity of the included studies. While the high proportion of positive results in this systematic review is encouraging, we are unable to rule out the possibility of publication bias, in which studies with a negative result are not published. Furthermore, our strict inclusion criteria led to the exclusion of several studies that lacked the relevant control groups but which could still provide valuable insight on this topic. While this highlighted inconsistencies in the quality of preclinical studies, further systematic reviews with less strict inclusion criteria could be a useful supplement to this review to explore PTB models in other species.

## Conclusion

This is the first systematic review of prenatal interventions for infection/inflammation-induced PTB in preclinical models. This review identifies several interventions and potential inflammatory targets in mice that hold promise for clinical translation and warrant further investigation. However, the heterogeneous nature of the PTB models and poor reporting of methodological design highlights the need for standardised protocols for the undertaking and reporting of preclinical studies.

## Supplementary Information

Below is the link to the electronic supplementary material.

Supplementary file1 (DOCX 31 KB)

Supplementary file2 (DOCX 89 KB)

## Data Availability

All data is included in the Tables and Supplementary Files provided with this manuscript.

## Code Availability

No code was used nor generated in this study.
