# Supplementary material for: Interventions for Infection and Inflammation-Induced Preterm Birth: a Preclinical Systematic Review
Source: Reprod Sci. 2022 Apr 14;30(2):361–79. doi: 10.1007/s43032-022-00934-x (PMC9988807; doi:10.1007/s43032-022-00934-x)
Supplement: Supplementary file 1 — Supplementary file1 (DOCX 31 KB) [file 43032_2022_934_MOESM1_ESM.docx]

**Title**

Interventions for infection and inflammation-induced preterm birth: a preclinical systematic review

**Journal**

Reproductive Sciences

**Authors**

Ms Faith A MILLER^1^, Dr Adalina SACCO^1^, Professor Anna L DAVID^1, 2^, Dr Ashley K BOYLE^1^

^1^ Elizabeth Garrett Anderson Institute for Women’s Health, University College London, London, UK

^2^ National Institute for Health Research University College London Hospitals Biomedical Research Centre, London, UK

**Corresponding author**

Dr Ashley K Boyle [Ashley.boyle@ucl.ac.uk](mailto:Ashley.boyle@ucl.ac.uk)

**Supplementary Table 1** Search terms used 19/06/2020 and repeated 20/01/2021.

| Terms applied to MEDLINE search | | | | | |
| --- | --- | --- | --- | --- | --- |
| Concept | Terms | | | Conjunctive | |
| Animal Models | “Models, Animal”[MeSH] | | | Combine with "OR" | Combine with "AND" |
|  | Animal |  |  |  |  |
|  | Bovine |  |  |  |  |
|  | Canine |  |  |  |  |
|  | Cat |  |  |  |  |
|  | Cow |  |  |  |  |
|  | Dog |  |  |  |  |
|  | Equine |  |  |  |  |
|  | Feline |  |  |  |  |
|  | Guinea pig |  |  |  |  |
|  | Horse |  |  |  |  |
|  | In Vivo |  |  |  |  |
|  | Marsupial |  |  |  |  |
|  | Mice |  |  |  |  |
|  | Mouse |  |  |  |  |
|  | Pig |  |  |  |  |
|  | Rabbit |  |  |  |  |
|  | Rat |  |  |  |  |
|  | Rhesus Macaque | | |  |  |
|  | Monkey |  |  |  |  |
|  | Sheep |  |  |  |  |
| Preterm birth | “Premature Birth”[MeSH] | | | Combine with "OR" |  |
|  | Preterm | "AND" | Birth |  |  |
|  | Premature |  | Labour |  |  |
|  | Pre-term |  | Labor |  |  |
|  |  |  | Delivery |  |  |
|  |  |  | Pregnan*  Parturition |  |  |
| Inflammation | Inflammation[MeSH] | |  | Combine with "OR" |  |
|  | “Bacteria”[MeSH] | | |  |  |
|  | Infect* |  |  |  |  |
|  | Inflamm* |  |  |  |  |
|  | Chorioamnionitis | | |  |  |
|  | Bacteria* |  |  |  |  |
|  | Cytokine |  |  |  |  |
|  | Viral |  |  |  |  |
|  | Virus |  |  |  |  |
|  | Microb* |  |  |  |  |
| Therapeutics | “Therapeutics”[MeSH] | | | Combine with "OR" |  |
|  | Therap* |  |  |  |  |
|  | Treat* |  |  |  |  |
|  | Intervention* |  |  |  |  |
|  | Prevent* |  |  |  |  |

| Terms applied to EMBASE search | | | | | | | | | |  |
| --- | --- | --- | --- | --- | --- | --- | --- | --- | --- | --- |
| Concept | Terms | | | | | Conjunctive | | | |  |
| Animal Models | exp *Animal model/ | | | | | Combine with "OR" | | Combine with "AND" | |  |
|  | Animal |  | |  | |  |  |  |  |  |
|  | In vivo |  | |  | |  |  |  |  |  |
|  | Mouse |  | |  | |  |  |  |  |  |
|  | Mice |  | |  | |  |  |  |  |  |
|  | Rat |  | |  | |  |  |  |  |  |
|  | Rabbit |  | |  | |  |  |  |  |  |
|  | Sheep |  | |  | |  |  |  |  |  |
|  | Cow |  | |  | |  |  |  |  |  |
|  | Bovine |  | |  | |  |  |  |  |  |
|  | Horse |  | |  | |  |  |  |  |  |
|  | Equine |  | |  | |  |  |  |  |  |
|  | Dog |  | |  | |  |  |  |  |  |
|  | Canine |  | |  | |  |  |  |  |  |
|  | Cat |  | |  | |  |  |  |  |  |
|  | Feline |  | |  | |  |  |  |  |  |
|  | Pig |  | |  | |  |  |  |  |  |
|  | Guinea Pig |  | |  | |  |  |  |  |  |
|  | Rhesus Macaque | | | | |  |  |  |  |  |
|  | Monkey |  | |  | |  |  |  |  |  |
|  | Marsupial |  | |  | |  |  |  |  |  |
| Preterm Birth | exp *Premature labor/ | | | | | Combine with "OR" | |  |  |  |
|  | exp *"Immature and premature labor"/ | | | | |  |  |  |  |  |
|  | Preterm | “and” | | Birth | |  |  |  |  |  |
|  | Premature |  |  | Labour | |  |  |  |  |  |
|  | Pre-term |  |  | Labor | |  |  |  |  |  |
|  |  |  |  | Delivery | |  |  |  |  |  |
|  |  |  |  | Pregnan*  Parturition | |  |  |  |  |  |
| Inflammation | exp *Inflammation/ | | | | | Combine with "OR" | |  |  |  |
|  | exp *Chorioamnionitis/ | | |  | |  |  |  |  |  |
|  | Inflamm* |  | |  | |  |  |  |  |  |
|  | Bacteria* |  | |  | |  |  |  |  |  |
|  | Cytokine |  | |  | |  |  |  |  |  |
|  | Viral |  | |  | |  |  |  |  |  |
|  | Virus |  | |  | |  |  |  |  |  |
|  | Microb* |  | |  | |  |  |  |  |  |
|  | Infect* | | | | |  |  |  |  |  |
| Therapeutics | exp *Intervention study/ | | | | | Combine with "OR" | |  |  |  |
|  | exp *biological therapy/ | | | | |  |  |  |  |  |
|  | exp *therapy/ |  | |  | |  |  |  |  |  |
|  | Therap* |  | |  | |  |  |  |  |  |
|  | Treat* |  | |  | |  |  |  |  |  |
|  | Intervention* |  | |  | |  |  |  |  |  |
|  | Prevent* |  | |  | |  |  |  |  |  |
| Terms applied to Web of Science search | | | | | | | | | | |
| Concept | Terms | | | | | | Conjunctive | | | |
| Animal Models | Animal | |  | |  | | Combine with "OR" | | Combine with "AND" | |
|  | In vivo | |  | |  | |  |  |  |  |
|  | Mice | |  | |  | |  |  |  |  |
|  | Rat | |  | |  | |  |  |  |  |
|  | Rabbit | |  | |  | |  |  |  |  |
|  | Sheep | |  | |  | |  |  |  |  |
|  | Cow | |  | |  | |  |  |  |  |
|  | Bovine | |  | |  | |  |  |  |  |
|  | Horse | |  | |  | |  |  |  |  |
|  | Equine | |  | |  | |  |  |  |  |
|  | Dog | |  | |  | |  |  |  |  |
|  | Canine | |  | |  | |  |  |  |  |
|  | Cat | |  | |  | |  |  |  |  |
|  | Feline | |  | |  | |  |  |  |  |
|  | Guinea pig | |  | |  | |  |  |  |  |
|  | Pig | |  | |  | |  |  |  |  |
|  | Rhesus Macaque | |  | |  | |  |  |  |  |
|  | Monkey | |  | |  | |  |  |  |  |
|  | Marsupial | |  | |  | |  |  |  |  |
| Preterm birth | Premature | | "AND" | | Birth | | Combine with "OR" | |  |  |
|  | Preterm | |  |  | Labour | |  |  |  |  |
|  | Pre-term | |  |  | Labor | |  |  |  |  |
|  |  | |  |  | Delivery | |  |  |  |  |
|  |  | |  |  | Pregnan*  Parturition | |  |  |  |  |
| Inflammation | Infect* | |  | |  | | Combine with "OR" | |  |  |
|  | Inflamm* | |  | |  | |  |  |  |  |
|  | Bacteria* | |  | |  | |  |  |  |  |
|  | Cytokine | |  | |  | |  |  |  |  |
|  | Viral | |  | |  | |  |  |  |  |
|  | Virus | |  | |  | |  |  |  |  |
|  | Microb* | |  | |  | |  |  |  |  |
|  | Chorioamnionitis | |  | |  | |  |  |  |  |
| Therapeutics | Therap* | |  | |  | | Combine with "OR" | |  |  |
|  | Treat* | |  | |  | |  |  |  |  |
|  | Intervention* | |  | |  | |  |  |  |  |
|  | Prevent* | |  | |  | |  |  |  |  |
