# Supplementary material for: Interventions for Infection and Inflammation-Induced Preterm Birth: a Preclinical Systematic Review
Source: Reprod Sci. 2022 Apr 14;30(2):361–79. doi: 10.1007/s43032-022-00934-x (PMC9988807; doi:10.1007/s43032-022-00934-x)
Supplement: Supplementary file 2 — Supplementary file2 (DOCX 89 KB) [file 43032_2022_934_MOESM2_ESM.docx]

**Title**

Interventions for infection and inflammation-induced preterm birth: a preclinical systematic review

**Journal**

Reproductive Sciences

**Authors**

Ms Faith A MILLER^1^, Dr Adalina SACCO^1^, Professor Anna L DAVID^1, 2^, Dr Ashley K BOYLE^1^

^1^ Elizabeth Garrett Anderson Institute for Women’s Health, University College London, London, UK

^2^ National Institute for Health Research University College London Hospitals Biomedical Research Centre, London, UK

**Corresponding author**

Dr Ashley K Boyle [Ashley.boyle@ucl.ac.uk](mailto:Ashley.boyle@ucl.ac.uk)

**Supplementary Table 1** Data extraction

| 1. Publication details | | | | |
| --- | --- | --- | --- | --- |
| Paper | Authors | Year of publication | Institution | Journal |
| Schmitz *et al.*, 2007 | Thomas Schmitz, Evelyne Souil, Roxane Herve´, Carole Nicco,Fre´de´ric Batteux, Guy Germain, Dominique Cabrol,Danie`le Evain-Brion, Marie-Jose`phe Leroy, and Ce´line Me´hats | 2007 | Institut National de la Sante´ et de la Recherche Me´dicale, Unite´ 767, Paris, France | The Journal of Immunology |
| Peltier *et al*., 2013 | Morgan R. Peltier, Hschi-Chi Koo, Ellen M. Gurzenda, Yuko Arita, Natalia G. Klimova, Niccole Olgun, Nazeeh Hanna | 2013 | Winthrop University Hospital, Mineola, NY, USA | American Journal of Reproductive Immunology |
| Sykes *et al.*, 2012 | Lynne Sykes, Bronwen R. Herbert, David A. MacIntyre, Emma Hunte, Sathana Ponnampalam, Mark R. Johnson, Tiong G. Teoh and Phillip R. Bennett | 2012 | Imperial College London | Immunology |
| Domínguez Rubio *et al.*, 2014 | Domínguez Rubio, A. P. D.; Sordelli, M. S.; Salazar, A. I.; Aisemberg, J.; Bariani, M. V.; Cella, M.; Rosenstein, R. E.; Franchi, A. M. | 2014 | University of Buenos Aires/CONICET | Journal of Pineal Research |
| Shynlova *et al.*, 2014 | Shynlova, O.; Dorogin, A.; Li, Y. Q.; Lye, S. | 2014 | Research Institute at Mount Sinai Hospital | Journal of Cellular and Molecular Medicine |
| Yang *et al.*, 2014 | Yang, S. W.; Li, W.; Challis, J. R. G.; Reid, G.; Kim, S. O.; Bocking, A. D. | 2014 | University of Toronto Faculty of Medicine | American Journal of Obstetrics and Gynecology |
| Filipovich *et al.*, 2015 | Filipovich, Y.; Agrawal, V.; Crawford, S. E.; Fitchev, P.; Qu, X. W.; Klein, J.; Hirsch, E. | 2015 | Pritzker School of Medicine, | American Journal of Obstetrics and Gynecology |
| Chin *et al*., 2016 | Chin, P. Y.; Dorian, C. L.; Hutchinson, M. R.; Olson, D. M.; Rice, K. C.; Moldenhauer, L. M.; Robertson, S. A. | 2016 | University of Adelaide | Scientific reports |
| Liu *et al*., 2016 | Liu, W. N.; Xu, C.; You, X. J.; Olson, D. M.; Chemtob, S.; Gao, L.; Ni, X. | 2016 | Second Military Medical University, Shanghai, | PLoS One |
| Lei *et al*., 2017 | Lei, J.; Rosenzweig, J. M.; Mishra, M. K.; Alshehri, W.; Brancusi, F.; McLane, M.; Almalki, A.; Bahabry, R.; Arif, H.; Rozzah, R.; Alyousif, G.; Shabi, Y.; Alhehaily, N.; Zhong, W. Y.; Facciabene, A.; Kannan, S.; Kannan, R. M.; Burd, I. | 2017 | Johns Hopkins University School of Medicine, Baltimore, MD | Scientific reports |
| Madaan *et al.*, 2017 | Madaan, A.; Nadeau-Vallee, M.; Rivera, J. C.; Obari, D.; Hou, X.; Sierra, E. M.; Girard, S.; Olson, D. M.; Chemto, S. | 2017 | McGill University, Montre´ al, Que´ bec | American Journal of Obstetrics and Gynecology |
| Rinaldi *et al.*, 2015 | Rinaldi, S. F.; Catalano, R. D.; Wade, J.; Rossi, A. G.; Norman, J. E. | 2015 | University of Edinburgh | Molecular Human Reproduction |
| Arenas-Hernandez *et al.*, 2019 | Arenas-Hernandez, M.; Romero, R.; Xu, Y.; Panaitescu, B.; Garcia-Flores, V.; Miller, D.; Ahn, H.; Done, B.; Hassan, S. S.; Hsu, C. D.; Tarca, A. L.; Sanchez-Torres, C.; Gomez-Lopez, N. | 2019 | Wayne State University School of Medicine, Detroit, MI | Journal of Immunology |
| Boyle *et al.*, 2019 | Boyle, A. K.; Rinaldi, S. F.; Rossi, A. G.; Saunders, P. T. K.; Norman, J. E. | 2019 | University of Edinburgh | FASEB Journal |
| Herbert *et al.*, 2019 | Herbert, B. R.; Markovic, D.; Georgiou, E.; Lai, P. F.; Singh, N.; Yulia, A.; Johnson, M. R. | 2019 | Imperial College, London | Biology of Reproduction |
| Nadeau-Vallée *et al*., 2017 | Mathieu Nadeau-Vallée, Peck-Yin Chin, Lydia Belarbi, Marie-Ève Brien, Sheetal Pundir, Martin H. Berryer, Alexandra Beaudry-Richard, Ankush Madaan, David J. Sharkey, Alexis Lupien-Meilleur, Xin Hou, Christiane Quiniou, Alexandre Beaulac, Ines Boufaied, Amarilys Boudreault, Adriana Carbonaro, Ngoc-Duc Doan, Jean-Sebastien Joyal, William D. Lubell, David M. Olson, Sarah A. Robertson, Sylvie Girard and Sylvain Chemtob | 2017 | CHU Sainte-Justine Research Center, Montreal, Quebec | The Journal of Immunology |
| Schander *et al*., 2020 | Schander, J. A.; Aisemberg, J.; Correa, F.; Wolfson, M. L.; Juriol, L.; Cymeryng, C.; Jensen, F.; Franchi, A. M. | 2020 | Centro de Estudios Farmacológicos y Botánicos, CONICET-UBA, Buenos Aires, Argentina | Reproduction |
| Wahid *et al.*, 2020 | Wahid, H. H.; Chin, P. Y.; Sharkey, D. J.; Diener, K. R.; Hutchinson, M. R.; Rice, K. C.; Moldenhauer, L. M.; Robertson, S. A. | 2020 | University of Adelaide | American Journal of Pathology |
| Chen *et al*., 2012 | Chen, Y. H.; Zhao, M.; Chen, X.; Zhang, Y.; Wang, H.; Huang, Y. Y.; Wang, Z.; Zhang, Z. H.; Zhang, C.; Xu, D. X. | 2012 | Anhui Medical University | J Immunol |
| Toda *et al.*, 2016 | Toda, A.; Sawada, K.; Fujikawa, T.; Wakabayashi, A.; Nakamura, K.; Sawada, I.; Yoshimura, A.; Nakatsuka, E.; Kinose, Y.; Hashimoto, K.; Mabuchi, S.; Tokuhira, A.; Nakayama, M.; Itai, A.; Kurachi, H.; Kimura, T. | 2016 | Osaka University Graduate School of Medicine, Osaka | Am J Pathol |
| Agrawal *et al*., 2018 | Agrawal, V.; Jaiswal, M. K.; Beaman, K. D.; Hirsch, E. | 2018 | NorthShore University HealthSystem, Evanston, Illinois,USA | Biol Reprod |
| Fu *et al.*, 2019 | Fu, L.; Chen, Y. H.; Xu, S.; Yu, Z.; Zhang, Z. H.; Zhang, C.; Wang, H.; Xu, D. X. | 2019 | Anhui Medical University, Hefei 230032, China | International Immunopharmacology |
| Zhang *et al.*, 2020 | Zhang, J.; Luo, X.; Huang, C.; Pei, Z.; Xiao, H.; Luo, X.; Huang, S.; Chang, Y | 2020 | Guangdong Women and Children Hospital, Guangzhou, | Am J Reprod Immunol |

| 1. PTB model details | | | | | | | | | | |
| --- | --- | --- | --- | --- | --- | --- | --- | --- | --- | --- |
| Paper | Species | Sample size | Animal strain | Agent used | Dosage | Preparation | Administration route | Control used | GA at PTB induction (days ) | Plug day designation |
| Schmitz *et al.*, 2007 | Mice | 11-13 | CD-1 | LPS (O127:B8) | 10ug LPS | in 100ul PBS | Injection between gestational sacs of left uterine horn | 100uL PBS | GD15 | N/A |
| Peltier *et al*., 2013 | Mice | 10-21 | CD-1 | E.coli (serotype N/S) | 10^6 CFU E.coli (100uL) | in 100ul PBS | Laparotomy and injection between gestational sacs | 100uL PBS | GD14 | N/A |
| Sykes *et al.*, 2012 | Mice | 4 | CD-1 | LPS  (0111:B4) | 20ug | 25uL total volume | Mini-laparotomy and injection into upper right uterine horn between the first and second sacs | PBS | E16 | E0 |
| Domínguez Rubio *et al.*, 2014 | Mice | 10 | BALB/c | LPS (serotype N/S) | 2 doses: 10ug (0.26mg/kg) at 10am 20ug (0.52mg/kg) at 1pm | in 100uL sterile saline | I.P. injection | 100uL sterile saline | GD15 | GD0 |
| Shynlova *et al.*, 2014 | Mice | 6-11 | CD-1 | LPS (055:B5) | 50ug LPS | in 100ul sterile saline | I.P. injection | 100ul sterile saline | GD15 | GD0.5 |
| Yang *et al.*, 2014 | Mice | LPS dose studies =10 PTB studies =9-17 Chemokine studies =10 | CD-1 | LPS (055:B5) | 125ug used | in 100uL saline | mini-laparotomy and injection into lowers segment of uterine horns - between 2 lowest gestational sacs of left or right horn | 100uL saline | GD15 | GD1 |
| Filipovich *et al.*, 2015 | Mice | 4-10 | CD1 | E.coli (serotype N/S) | 2x10^7 or 6x10^7 heat-killed E coli | in 100uL PBS | Mini-laparotomy and IU injection in midsection of right uterine horn on side between 2 adjacent fetuses | 100uL PBS | GD14.5 + 5hrs | GD0.5 |
| Chin *et al*., 2016 | Mice | 10-20 | C57BL/6 (B6) | LPS (S. typhimurium)   OR E.coli (o55:K59(B5):H-) | 0.5ug LPS - Dose to elicit 50% PTD  OR   5 × 10^10 cfu in 100 μ l E.coli - also elicit 50% PTD | in 100/200ul PBS | LPS: I.P. injection  E.coli: injected into midsection of left uterine horn between 2 adjacent fetuses | 200uL PBS | GD16.5 (at 11.00h) | GD0.5 (checked 08-10h for plug) |
| Liu *et al*., 2016 | Mice | 6-12 | BALB/c | LPS (0111:B4) | 0.2, 0.4 or 0.8mg/kg - 0.4 chosen for NaHS studies | in 100ul normal saline (NS) | 2 I.P. injections over 3 hours | 100ul NS | GD14.5 | GD0.5 (checked 6am) |
| Lei *et al*., 2017 | Mice | 4-72 | CD-1 | LPS (O55:B5) | 25ug | in 100ul PBS | laparotomy and injected in between first and second embryos of right uterine horn | 100uL PBS | GD17 | N/A |
| Madaan *et al.*, 2017 | Mice | 4-22 | CD-1 | LPS (serotype N/S) | 10ug LPS | in 100uL saline | I.P. injection | 100uL saline | GD16 | N/A |
| Rinaldi *et al.*, 2015 | Mice | 44174 | CD-1 | LPS (0111:B4) | 20ug | 25uL PBS | mini-laparotomy - inject uterine horn with highest #fetuses in, between 1st and 3nd anterior fetuses | 25uL sterile PBS | GD17 | GD1 |
| Paper | Species | Sample size | Animal strain | Agent used | Dosage | Preparation | Administration route | Control used | GA at PTB induction (days ) | Plug day designation |
| Arenas-Hernandez *et al.*, 2019 | Mice | 5-10 | C57BL/6J | Anti-CD3ε Ab | 10ug/200ul monoclonal anti-CD3e Ab | 200ul PBS | I.P. injection | 10 mg/200 ml of IgG1 k isotype control in PBS | GD16.5 | GD0.5 |
| Boyle *et al.*, 2019 | Mice | 10-25 | C56BL/6J | LPS (0111:B4) | 1ug | in 25ul PBS | IU injection into uterine lumen between 2 gestational sacs | 25ul PBS | GD17 | GD1 |
| Herbert *et al.*, 2019 | Mice | 5-14 | CD-1 | LPS (0111: B4) | 10ug LPS in 25ul PBS | in 25ul PBS | mini laparotomy and injection on right uterine horn between 1st and 2nd uppermost fetuses | 25ul PBS | GD16 | GD0 |
| Nadeau-Vallée *et al*., 2017 | Mice | 10 | C57BL/6 (B6) | LPS (S. typhimurium) | 0.5ug LPS | in 200ul PBS | Injection between fetal membrane in lower segment of uterine horn | PBS | GD16.5 (at 10.00-1200h) | GD0.5 |
| Schander *et al*., 2020 | Mice | 16 | BALB/c | LPS (05:B55) | 2 doses: 1st: 0.13mg/kg and 2nd: 0.39mg/kg | saline solution | I.P. injection | sterile saline (SS) | GD15: 1st 9am, 2nd 12pm | GD0 |
| Wahid *et al.*, 2020 | Mice | 6-10 | BALB/c | Carbamyl-Platelet Activating Factor (cPAF) | 2ug/mouse for I.P.  35ug/mouse for I.U. | in 100ul PBS | I.U. injection in right uterine horn between 2 adjacent fetuses closes to the cervix or I.P. | 100 ulPBS | GD16.5 (10am-12pm) | GD0.5 |
| Chen *et al*., 2012 | Mice | 11 | ICR | LPS (0127:B8) | 1 OR 3 doses of 75ug/kg (GD15 OR 15-17) | normal saline (NS) | I.P. injection | NS | Uterine/fetal weight, fetal survival & crown-rump length: daily injections GD15-17  Placental COX-2, cytokine & placenta, liver MT1&2 expression: 1 injection GD15 | GD0 |
| Toda *et al.*, 2016 | Mice | 6-22 | C3H/HeN | LPS (055:B05) | 2x dose of 50ug/kg LPS | in 200uL 0.9% saline | I.P. injection | PBS | GD15.5: 2 injections with 3hr interval; 2pm & 5pm | GD0.5 |
| Agrawal *et al*., 2018 | Mice | 3-15 | CD-1 | E.coli (ATCC No. 12014) | 1–3 × 103 E. coli organisms | 20ul, made up to 100ul with either SPA or saline | I.U. injection in midsection of right uterine horn between 2 adjacent fetuses | sterile control solution | GD14.5 | N/A |
| Fu *et al.*, 2019 | Mice | 15-20 | ICR | LPS (0127: B8) | 200ug/kg | in normal saline | I.P. injection | NS | GD15 | GD0 |
| Zhang *et al.*, 2020 | Mice | 10-14 | BALB/c | LPS (0111:B4) | 2 x injection of 50ug/kg | in 200ul normal saline | I.P. injection | NS | GD15 (2 injections 3 hours apart) | GD0 |

| 1. Therapy details | | | | | | | |
| --- | --- | --- | --- | --- | --- | --- | --- |
| Paper | Agent used | Dosage | Preparation | Control used | Administration route | Timing of administration (relative to PTB induction) | Duration of Intervention |
| Schmitz *et al.*, 2007 | Rolipram (phosphodiesterase-4 inhibitor) | 3mg/kg | in 100ul PBS | 5%DMSO in PBS | I.P. injection | 2hrs after LPS/PBS | Single injection |
| Peltier *et al*., 2013 | Carbon monoxide (CO)-saturated lactated Ringer’s solution (LRS) | CO saturated into LRS to concentration of 800ppm | [C0] 800ppm in LRS | 1ml LRS | I.P. injection | 1-3hrs before E.coli/PBS, and then daily until delivery/euthanised on GD15 | I.P. injection daily until delivery |
| Sykes *et al.*, 2012 | Pyl A (CRTH2 (chemokine receptor expressed on Th2 lymphocytes) agonist) | 250ug Pyl A |  | 250ug vehicle | IU injection between 2nd and 3rd sacs | Same time as LPS/vehicle | Single injection |
| Domínguez Rubio *et al.*, 2014 | Melatonin | 25mg melatonin | In pellet with 3% w/v vegetable oil compressed in metallic punch | Sham operated without pellet implantation | S.C. implant | GD14 (day before LPS) | Single operation to implant pellet |
| Shynlova *et al.*, 2014 | BSCI (Broad Spectrum Chemokine Inhibitor) | 10mg/kg | in 100ul corn oil/ethanol | corn oil/ethanol | subcutaneous injection | 2 injections: GD14 (24 hrs before LPS) and GD15 (prior to LPS). Then daily injections until delivery | GD14-delivery |
| Yang *et al.*, 2014 | L rhamnosus GR-1 | 20uL GR1 supernatant (cont. 2 x 10^7 - 10^8 colony forming units/mL of bateria) | GR1 grown in supernatant | 200uL saline | I.P. injection | 2 doses: 24hr pre LPS (GD14) 30 minutes before LPS (GD15) | 2 doses over 23.5hrs |
| Filipovich *et al.*, 2015 | rabit polyclinal antimouse polymorphonuclear leukocytes (PMN) antiserum | 0.5mL total; 1:10 in PBS | in PBS | 2 controls: Conrol rabbit serum (diluted 1:10 in PBS) PBS | I.P. injection | 2 I.P. injections: 29hrs and 5hs before E.coli/PBS (GD 13.5 & 14.5) | 2 doses over 24hrs |
| Chin *et al*., 2016 | rabit polyclinal antimouse polymorphonuclear leukocytes (PMN) antiserum | 60mg/kg | in 100ul PBS + 0.1% BSA | 100ul PBS + 0.1% BSA | I.P. injection | 4 doses: GD16.5 within 5 min of LPS Every 12hr after (GD17, 17.5 &18) | 4 doses over 36hrs |
| Liu *et al*., 2016 | hydrogen sulfide (H2S) (using sodium hydrosulfide, NaHS, as a generator of H2S in biological tissues) | 5, 7.5, 10, 15 mg/kg  10mg/kg used | in 100ul NS | 100ul NS | I.P. injection | 2 injections over 3 hours; same time as LPS/NS at 8.30 or 11.30am | 2 doses over 3 hours |
| Lei *et al*., 2017 | dendrimer nanoparticle N-acetyl cysteine (DNAC)  (Also NAC and Cy5-labeled dendrimer but wrong controls) | 150uL DNAC  (150uL NAC, 150uL Cy5) | 10mg/kg  (NAC 10mg/kg or 100mg/kg and 10mg/kg Cy5) | 150ul PBS | I.P. injection | GD17 - 1hr post LPS | 1 injection |
| Madaan *et al.*, 2017 | LV.shGPR81  Also: agonist of GPR81: 3,5-DHBA | LV.shGPR81: 100uL total (50uL to each horn)  25mg/kg/8hrs of 3,5-DHBA | LV vector  100ul; 'vehicle' for DHBA not clear | vehicle or LV.shGFP | LV: IU injection (lower segment of both uterine horns between 2 fetal membranes)  DHBA: subcutaneous injection (neck) | LV: GD13 (4 days before LPS)  DHBA: 30 mins before LPS | 1 injection timepoint |
| Rinaldi *et al.*, 2015 | 15-epi-lipoxin A4 | 12.5 or 125ng | in 100il PBS | 100ul PBS + 1% ethanol | I.P. injection | 1-2 hours pre-LPS | 1 injection |
| Paper | Agent used | Dosage | Preparation | Control used | Administration route | Timing of administration (relative to PTB induction) | Duration of Intervention |
| Arenas-Hernandez *et al.*, 2019 | p4 | 1mg/100ul | sesame oil | 100ul SO | S.C. injection | GD15.5, 16.5 and 17.5 | 3 injections over 48hrs |
| Boyle *et al.*, 2019 | Simvastatin | 20 or 40ug | PBS | ? Volume PBS | I.P. injection | GD16 and GD17 (24h before LPS and 2hr after LPS) | 2 inections over 26hr |
| Herbert *et al.*, 2019 | Aminophylline (Am), a cAMP-PDE inhibitor and p4 | 10mg/kg Am  5mg P4 | Am in DMSO  p4 in peanut oil | AM: DMSO  P4: peanut oil | Am: I.P. injection  P4: S.C. injection | All GD16: Am: 120 min before LPS  P4: 30 min before LPS | 2 injection over 90 minute (single injection of each Am and P4) |
| Nadeau-Vallée *et al*., 2017 | small peptide non-competitive IL-1R antagonist (termed 101.10) | 1mg/kg per 12h | 100ul 101.1 | 100ul vehicle | I.P. injection | 30 mins before LPS/vehicle, and then every 12 hrs (GDs 16.5, 17, 17.5 & 18) | 4 injections over 36 hours |
| Schander *et al*., 2020 | Enriched environment (EE): bigger cage (640x420x200mm) with running wheels and objects of diff shapes, textures and colours which are changed once/week | N/A | N/A | standard condition (CE) | N/A | 6 weeks of EE before mating, and until GD15 (all mice 6 weeks old before starting EE) | 6 weeks of EE before mating, and until GD15 |
| Wahid *et al.*, 2020 | naltrexone (nx) TLR4 antagonist | 60mg/kg | in PBS | PBS | I.P. injection | 4 doses at 12h intervals: within 5 minutes of cPAF, GD17, 17.5 and 18 | 4 doses over 36hrs |
| Chen *et al*., 2012 | Zinc (Zn) | 75mg/litre in deionized water | in drinking water | deionized drinking water | oral | GD0-15 | GD0-15 |
| Toda *et al.*, 2016 | IMD-0560  or  MR16-1 (anti-mouse IL-6R antibody) | 30 mg/kg IMD-0560  or  12 mg/kg MR16-1 | in Vosco S-55  or  MR16-1 in 200uL | Vosco S-55 alone  or   nonimmune mouse IgG | Vosco S-55 transvaginal suppository  MR16-1 I.P. injection | Vosco S-55: 4hrs before 1st LPS (10am GD15.5) and with 2nd LPS (5pm GD15.5)  MR16-1: 24hr before LPS (5pm GD14.5) | Vosco S-55: 2 injections 7hrs apart  MR16-1: single injection |
| Agrawal *et al*., 2018 | SP-A | 100ug or 200ug | in water | medium | either: 1. IU  2. IV  3. IV | either: 1. IU with E.coli 2. IV same time as E.coli 3. IV 4h post E.coli | Single injection |
| Fu *et al.*, 2019 | vitamin D supplementation | 25ug/kg | in corn oil | corn oil | oral | once daily GD13-15 | duration not clear GD13-15 |
| Zhang *et al.*, 2020 | recombinant erythropoietin (EPO) | 20000 IU/kg | ? NS | NS | I.P. injection | 2x injections (1 hour before each LPS/NS injection) | 2 injections over 3 hrs |

| 1. Effect of the intervention on gestation length | | | | |
| --- | --- | --- | --- | --- |
| Paper | Effect of intervention of gestational length | PTB reported as | Definition of PTB | Time between model induction and outcome measurement |
| Schmitz *et al.*, 2007 | Saline-vehicle 1/12 preterm LPS-vehicle 11/13** (p<0.001 vs SV) Saline-rolipram 1/11  LPS-rolipram 2/13 (p<0.05 vs LPS) | Proportion of dam delivering preterm/total dams in each group | Delivery of >1 pup 48hrs after LPS/PBS | 48 hours |
| Peltier *et al*., 2013 | PBS/LRS 0/14 (0%) preterm (20 (19, 20)) PBS/CO 0/10 (0%) preterm (20 (19, 20)) E. coli/LRS 10/13 (77%)** preterm (16 (15, 20))** (p=0.001 vs control for both) E. coli/CO 4/21 (19%)** preterm (19 (15, 20))** (p=0.001/p=0.014 vs E.coli only) | Proportion of dams delivering preterm/total dams in each group & Median GA @ delivery (minimum, maximum) | Delivery before 19 days gestation | Until delivery |
| Sykes *et al.*, 2012 | Vehicle control = delivery 64.5 hr post injection 20ug LPS only = 7.7 hr (P < 0.001) 20ug LPS + 250ug Pyl A = 5.8 hr (mean)   10ug LPS = 14.7 hr 10ug LPS/ 500ugPyl = 8.7 hr post injection (P < 0.01, vs 10ug LPS only)  250 ug Pyl A alone = not induce labour 500 ug Pyl A alone = labour at 44.8 hr  None of the vehicle control-treated mice delivered preterm. | Hours until delivery post injection | Delivered sig. earlier than controls (hours) | Until delivery |
| Domínguez Rubio *et al.*, 2014 | Control: 0% preterm, 100% term  Melatonin: 0% preterm, 100% term LPS: 100% preterm, 0% term Melatonin + LPS: 50% preterm, 50% term (not sig) | % dams delivering term/preterm | Term=delivery during night of GD18/early morning GD19 The beginning of preterm delivery was defined by the delivery of the first pup. | Until delivery |
| Shynlova *et al.*, 2014 | LPS - 100% preterm, 0% term - 20 ± 4.8 hr Tx to Del LPS + BCSI - 64% preterm, 36% term - 45.2 ± 26.4 hr * (p<0.05 vs LPS) BCSI - 0% preterm, 100% term - 75 hr  Vehicle - 0% preterm, 100% term - 75 hr | % dams delivering term/preterm Mean time to delivery (±SEM?? Table does not specify but other figs use SEM) | Delivery = finding at least 1 pup in cage within 48hrs of LPS | Observed until delivery.  Term mice killed GD18.75 for #live pups, BW of pups and placental weight Preterm mice killed during PTD. |
| Yang *et al.*, 2014 | GR1 reduced PTB rate: GR & LPS 0% LPS - 94% (16/17) LPS+GR1 - 57% (8/14) (p=0.028) | % of dams delivering term/total | delivery of at least 1 pup within 48 hours of LPS/saline | Until delivery |
| Filipovich *et al.*, 2015 | No sig impact on PTB rates  E.coli 2x10^7 ~90% E.coli 2x10^7 + PMN ~70% (not sigf) E.coli 6x10^7 ~100% E.coli 6x10^7 + PMN ~100% | & of dams delivering term/total | 1 fetus in cage/lower vagina within 48hrs (checked twice daily) | Until delivery |
| Chin *et al*., 2016 | PBS = 0% (0/15) LPS only - 55% (11/20) - 2 alive, 9 dead (p<0.05) LPS & naloxone - 0% (0/14) (p<0.05 vs LPS) naloxone alone - 0% (0/14)  E.coli: 70% PTD (7/10) – av. 36hr reduction in GL (p<0.05) 0 reversed by naloxone: 0/10 deliver before GD18 (p<0.05). Naloxone alone has no effect. | dead & alive pups born prematurely/total pups | Delivery of dead/alive pups before GD18 | Mice killed at GD18.5 - uterus removed |
| Paper | Effect of intervention of gestational length | PTB reported as | Definition of PTB | Time between model induction and outcome measurement |
| Liu *et al*., 2016 | NS: 124.38 ± 6.76 hours LPS 0.4 + NS : 7.67 ± 0.89 LPS 0.4 + NaHS 5 : 7.58 ± 0.53 LPS 0.4 + NaHS 7.5 : 9.21 ± 1.26 (p<0.05 v LPS+NS) LPS 0.4 + NaHS 10 : 16.75 ± 5.95 (p<0.05 v LPS+NS) LPS 0.4 + NaHS 15 : 7.88 ± 0.41 NaHS alone - no sig effect on labour | injection-to-delivery interval (hours) | Not defined, just time to delivery | until delivery |
| Lei *et al*., 2017 | Preterm Birth  PBS 0/12 (0.0%) LPS 54/72 (75%) PBS + DNAC 0/4 (0%) LPS + DNAC 16/37 (43.2%)p = 0.004 v LPS+PBS LPS + dendrimer 4/7 (57.1%) - not sig LPS + NAC10 8/12 (66.7%) - not sig LPS + NAC100 13/20 (65.0%) - not sig | Proportion of dams delivering preterm | deliveries pre GD19 | Mice observed for 32hrs post-surgery |
| Madaan *et al.*, 2017 | No sig effect of LV.shGPR81 on LPS induced PTD or in negative control group DHBA:DHBA sig prevented LPS induced PTB (p<0.001) from ~35% in LPS to ~70% [inferred from graph] in LPS/DHBA | Gestational duration post LPS (hrs; mean ± SEM) | Deliveries before 50hrs post-LPS | Until delivery |
| Rinaldi *et al.*, 2015 | LPS sig reduces time to delivery (LPS:27.54 hr + SEM 6.33; vs vehicle: 55.40 h + SEM 6.40 (p=0.001).  No sig difference with ELA4 (12.5ng: 27.02+4.57, 125: 26.82+2.61 (both still sig lower than vehicle p<0.01)) | Time to delivery (h) | delivery of the first pup within 36 h of intrauterine injection | Until delivery |
| Arenas-Hernandez *et al.*, 2019 | p4 sig prevent % increase in preterm: SO + isotype = 0% SO + anti-CD3e = 83.3% (p=0.01 vs") P4 + isotype = 0% p4 + anti-CD3e = 0% (p<0.001 vs anti-CD3e alone) Mean time to 1st pup was sig reduced in SO+anti-CD3e, and sig restored in p4 + anti-CD3e (p<0.001) | Rate of preterm and Time between plug designation to observation of 1st pup in cage bedding | delivery <18.0 dpc. | until delivery |
| Boyle *et al.*, 2019 | Time to delivery LPS: 29.74 ± 3.6 Simvastatin (20 mg) + LPS: 46.86 ± 6.5* - sig increase p<0.05 Simvastatin (40 mg) + LPS: 45.3 ± 4.7*- sig increase p<0.05 PBS: 63.54 ± 2.3 Simvastatin (20 mg) + PBS: 59.05 ± 2.2 Simvastatin (40 mg) + PBS: 52.84 ± 2.7 | Time from LPS/PBS to delivery of 1st pup (h) ± (SEM or SD - not clear) | None given | until delivery |
| Herbert *et al.*, 2019 | P4/Am/PBS - all 3 vehicle controls deemed the same by statistician: 65-80h to del LPS: 9.9h LPS/Am: 12.9h LPS/P4: 11.6h P4/Am/LPS: 74h (p<0.001 vs LPS) | Time from LPS/PBS to delivery | None given | until delivery |
| Nadeau-Vallée *et al*., 2017 | Inferred from graph Veh/veh ~ 19.9 veh/101.1 ~ 19.5 LPS/veh ~18.9 LPS/101.1 ~19.7 Sig reduction in GL in LPS/veh vs veh/veh AND sig increase in GL in LPS/101.1 vs LPS/veh (p<0.05) | Measured gestation length | N/A | Until delivery |
| Paper | Effect of intervention of gestational length | PTB reported as | Definition of PTB | Time between model induction and outcome measurement |
| Schander *et al*., 2020 | LPS CE: 82.4% PTB LPS EE: 50% EE (p<0.05) SS CE/EE: 0% PTB | % of dams delivering term/total | Expulsion of a pup <GD18 | Until delivery |
| Wahid *et al.*, 2020 | I.P.: +nx protects mice from cPAF induced PTD (p<0.05) I.U.: +nx sig reduces %PTD (31% in cPAF+PBS to ~ % in cPAF+nx (p<0.05) | %dams delivering preterm | delivery of at least 1 pup within 48 hours of cPAF | until delivery |
| Chen *et al*., 2012 | Litters preterm: control: 0/11 Zn: 0/11 LPS: 4/11 * (P<0.05 vs control/zn) LPS+Zn: 0/11 * (P<0.05 vs LPS) | # dams delivering preterm/total # dams | Delivery of more than 1 pup before GD18 | dams sacrificed at GD18 |
| Toda *et al.*, 2016 | % Dam delivering PTD PBS: 0% IMD-0569: GL 19.5 ± 0.4 days LPS: 100% / GL 16.5 ± 0 LPS+IMD0560: 45% (Sig lower than LPS p<0.001) / GL 18.1 ± 0.3 days (P < 0.001) | % dams del PTD Mean days to delivery | Delivery <GD18.5 | until delivery |
| Agrawal *et al*., 2018 | Medium + medium/SP-A (200 μg IV/IU) 0%PTD   SPA/water IU simultaneous: Live E. coli + water: 100% Live E. coli + SP-A (100 μg): 33% Live E. coli + SP-A (200 μg): 0%* (p<0.0001) Live E. coli + depleted SP-A: 88%  IV (simultaneous): Live E. coli + water: 100% Live E. coli + SP-A (100 μg): 25%* (p=0.004) Live E. coli + SP-A (200 μg): 33%  IV (delayed 4 h): Live E. coli + water: 100% Live E. coli + SP-A (100 μg): 50% * (p=0.006) Live E. coli + SP-A (200 μg): 42% | % dams delivering preterm | Finding at least one fetus in the cage/lower vagina within 48hrs of surgery | 48hrs post-surgery |
| Fu *et al.*, 2019 | Control : 0% PTD VD3: 0% LPS: 60%⁎(p<0.05) LPS+VD3: 35%† (vs LPS; p<0.05) | % dams delivering preterm | delivery before GD19 | GD19 |
| Zhang *et al.*, 2020 | LPS: 100% del PTB LPS+EPO: 45.5% del PTD (< 0.0001 vs LPS) | % dams del <GD19 | Dam delivering before GD19 | until delivery/GD19 |

| 1. Effect of the intervention on neonatal/pup survival | | | | |
| --- | --- | --- | --- | --- |
| Paper | Live born pups / litter size | LBP reported as | Pup survival (if follow-up) | Pup survival reported as |
| Schmitz *et al.*, 2007 | Saline-vehicle 8.7 ± 1.1 : 0.7 ± 0.3(11) LPS-vehicle 2.5 ± 2.5 : 7.5 ± 1.5 (2) Saline-rolipram 10.2 ± 1.5 : 0.9 ± 0.5* (10) (p<0.05) LPS-rolipram 7.4 ± 1.2 : 2.1 ± 0.6 (11) --> rolipram reduced fetal demise by 22% p<0.05 | Mean (±SEM) live pups per undelivered dam : dead pups per undelivered dam (# undelivered dam) | N/A | N/A |
| Peltier *et al*., 2013 | PBS/LRS 10 (4, 14) PBS/CO 11.5 (5, 15) E. coli/LRS 3 (p=0.001 v PBS/LRS) (1, 4) E. coli/CO 3.5 (3, 12) | Among dams having live births after 19 days gestation, shown are median number of pups (minimum, maximum) per litter | PN day 1 - relative proportions not noted, only n numbers PBS/LRS n=10 PBS/CO n=13 E.coli/LRS n=3 E. coli/CO n=10 | number of pups surviving |
| Sykes *et al.*, 2012 | LPS sig reduced fetal survival at 4.5 hr post injection to 20%; Pyl A sig restored to 100% (P<0.0001) BUT when allowed to labour spontaneously, no pups viable in LPS or LPS/PylA - not able to restore (p<0.0001 vs veh or Pyl only) | Mice were anaesthetized and underwent a caesarean section 4.5h post LPS. Fetuses were assessed for viability by assessment of colour and movement with or without mechanical stimulus. OR subgroup allowed to deliver spontaneously | N/A | N/A |
| Domínguez Rubio *et al.*, 2014 | Control: 10 ± 1 Melatonin: 9 ± 2 LPS: 0 Melatonin + LPS: 8 ± 2 (not sig) | mean live pups born/dam ± SEM | N/A | N/A |
| Shynlova *et al.*, 2014 | LPS - 12.3 LPS + BCSI - 12.1 No sig diff. BCSI - 14.5 Vehicle - 13.3 | Mean n pups/litter |  |  |
| Yang *et al.*, 2014 | No diff in litter size between mice delivering at term (or fetal weight) | Number liver pups in mice delivering at term | N/A | N/A |
| Filipovich *et al.*, 2015 | No diff in either measure | % fetus retained in utero at necroscopy (dead or alive) % retained fetus alive at necroscopy (48hrs post surgery; GD16.5) | N/A | N/A |
| Chin *et al*., 2016 | LPS = 58% reduction in viable fetuses vs PBS - reversed by naloxone (p<0.05)  E.coli 66% reduction in viable litter size at birth - reversed by naloxone (p<0.05) | Viable fetuses *in-utero* | Pup survival: 13% of E.coli pups born alive survived to 3 weeks (p<0.05 vs 90%/88% of PBS/naloxone controls), + naloxone rescued majority; with 56% surviving to 3 weeks (p<0.05) | Proportion of pups surviving to 3 weeks |
| Liu *et al*., 2016 | N/a | N/a | N/a | N/a |
| Lei *et al*., 2017 | N/A | N/A | N/A | N/A |
| Madaan *et al.*, 2017 | 3,5-DHBEA sig restores neonatal survival (dampened by LPS) p<0.001 - from around 20% to around 75% [inferred from graph] | % neonatal survival at birth assessed by counting breathing pups per litter at birth | N/A | N/A |
| Rinaldi *et al.*, 2015 | In all litters: In negative model control: ELA1 sig reduces proportion of dead pups (p<0.05). In LPS group: LPS sig increases proportion of dead pups(p<0.01) but ELA4 has no sig effect. In prem litters: In LPS: ELA4 sig. reduces proportion of dead pups in prem litters (0.55+0.12 versus 0.97+0.02 (LPS only), p<0.05) | Proportion of dead pups within 12-24hrs (#live/dead pups recorded ± SEM) | N/A | N/A |
| Paper | Live born pups / litter size | LBP reported as | Pup survival (if follow-up) | Pup survival reported as |
| Arenas-Hernandez *et al.*, 2019 | Neonatal mortality: SO + isotype = 5.7% SO + anti-CD3e = 83.3% (p<0.001 vs ") P4 + isotype = 10.9% p4 + anti-CD3e = 51.7% (p<0.001 vs anti-CD3e alone) | rate of neonatal mortality was calculated as the number of pups found dead among the total litter size | N/A | N/A |
| Boyle *et al.*, 2019 | %live born pups  LPS: 18.43 ± 7.6 Simvastatin (20 mg) + LPS: 40.39 ± 10.2 Simvastatin (40 mg) + LPS: 39.18 ± 9.1 PBS: 87.7 ± 4.7 Simvastatin (20 mg) + PBS: 88.29 ± 7.6 Simvastatin (40 mg) + PBS: 76.22 ± 7.6  simvastatin did not sig increase % live born pups (trend) | # pups found within 24 h of delivery / # viable pups counted via ultrasound on d 17. | N/A | N/A |
| Herbert *et al.*, 2019 | 7h post LPS: 100% control pups alive, LPS reduced to 40% (very sickly) - P4/Am/combo not able to restore.  At labour: 50% control pups alive, LPS reduced to 20% - P4/Am/combo not able to restore | Labour pup survival, and also 7hr post LPS (% of total pups) | N/A | N/A |
| Nadeau-Vallée *et al*., 2017 | Inferred from graph Veh/veh ~ 5.8 veh/101.1 ~ 6.2 LPS/veh ~3.9 * sig reduction (p<0.05) LPS/101.1 ~5.7 (trend to increase but p=0.058)  Newborn survival (%/dam) sig reduced by LPS (~60%, p<0.001), restored by 101.10 (100%, p<0.001) | Viable pups at birth - count/dam (means ± SEM) | Pup survival at 1 week: sig reduced by LPS (p<0.001), restored by 101.10 (p<0.001) | Pup survival determined by counting breathing and nonbreathing pups @ birth  After 1 week calculated out of viable pups at birth |
| Schander *et al*., 2020 | CE SS: 7 ± 1 (litter size) / 33.3% (%of mother that presented perinatal death)/ 24.3% (% dead pups) CE LPSL 7 ± 1 / 66.7% / 78.1% EE SS: 9 ± 1 / 16.7% / 16.7% EE LPS: 8 ± 1 / 20.0% / 16.7% Not noted any sig diffs | Litter size % of mothers delivering at least one dead pup % of dead pups within the litter that delivered at term (incl still birth and deaths PND1-7) | N/A | N/A |
| Wahid *et al.*, 2020 | I.P.: +nx restores % viable pregnancies, and % viable fetuses/dam to PBS groups (p<0.05). +nx restores GL and viable pups born/dam (p<0.05)  I.U.: +nx restores % viable pregnancies, and % viable fetuses/dam to PBS groups (p<0.05) | Number of viable pups born AND at GD18.5 # implantation sites and classified as viable (presence of live fetus and placenta) or not viable (anaemic, malformed, or severely growth-retarded fetus) AND gestation length | cPAF sig reduce % pup survival to 3 weeks, +nx sig restores it (p<0.05) | % pup survival to 3 week |
| Chen *et al*., 2012 | Live fetuses/dead fetus per litter control: 13.1 ± 1.5 / 0.8 ± 0.4  Zn:13.5 ± 0.9 / 0.3 ± 0.2 LPS: 10.1 ± 0.9 / 4.0 ± 1.0** (P<0.01 vs control/zn) LPS + Zn: 11.8 ± 0.7 / 1.5 ± 0.5 * (P<0.05 vs LPS) | Live fetuses/dead fetus per litter (mean ± SEM) | N/A | N/A |
| Toda *et al.*, 2016 | N/A | N/A | N/A | N/A |
| Paper | Live born pups / litter size | LBP reported as | Pup survival (if follow-up) | Pup survival reported as |
| Agrawal *et al*., 2018 | Medium + medium/SP-A (200 μg IV/IU) 100% fetuses alive in uterus at autopsy/delivery  PA/water IU simultaneous: Live E. coli + water: 0% Live E. coli + SP-A (100 μg): 66% Live E. coli + SP-A (200 μg): 91% Live E. coli + depleted SP-A: 6%  IV (simultaneous): Live E. coli + water: 0% Live E. coli + SP-A (100 μg): 42% Live E. coli + SP-A (200 μg): 73%  IV (delayed 4 h): Live E. coli + water: 0% Live E. coli + SP-A (100 μg): 48% Live E. coli + SP-A (200 μg): 57%  Sig (no p values given) improvement in fetal survival. NB no sig diff between 2 SP-A doses | % fetuses alive *in-utero* at autopsy/delivery (48hrs post E.coli)  survival status based on cardiac or vascular pulsations in the fetal bodies or membranes | N/A | N/A |
| Fu *et al.*, 2019 | Fetal death rate/litter: LPS sig increase (60.07%; p<0.01), VD3+LPS sig reduce (p<0.05) | Fetal death per litter after delivery | N/A | N/A |
| Zhang *et al.*, 2020 | CRL: 100% viable fetus / 8 ± 2 EPO: 100% / 7 ± 2 LPS: 0%* (P < 0.0001) / 0 LPS + EPO: 46%* (P < 0.0001) / 5 ± 1 | viable pup % number/total pups and viable pup number/mother | N/A | N/A |

| 1. Effect of the intervention on maternal inflammation | | | |
| --- | --- | --- | --- |
| Paper | Maternal inflammatory markers measured | Which tissue examined | Significant change in inflammatory marker? |
| Schmitz *et al.*, 2007 | TNFα, IL-1b, IL-6 and IL-10  uNK cells  Nuclear NFkB translocation | Amniotic fluid (TNFα, IL-1b, IL-6 and IL-10)   Decidual-placental tissue (uNK & NFkB nuclear translocation) | AF: LPS sig increase TNFα and IL-1b (p<0.05) and IL-6 and IL-10 (p<0.01) - Rolipram significantly blocked rise in TNFα, IL-1b, and IL-6, whereas it had no significant effect on IL-10 level. [NB no level of sig diff of LPS + rolipram from LPS given, just absence of difference from control]  Increase in inflammation induced mesometrial uNK recruitment seen in LPS, prevented in rolipram  LPS sig increase sustained NFkB nuclear translocation to the gestational tissues (p<0.01)- prevented by rolipram |
| Peltier *et al*., 2013 | IL-1B, TNFα, IL-6 and IL-10 - protein and mRNA expression | Amniotic fluid and placental tissue | E.coli sig increase AF [IL-1B] (p=0.001). No significant reduction in inflammation by CO (trend to reduce). Same for TNFα, IL-6, IL-10  E.coli sig increase IL-1B, IL-6, IL-10 levels in placenta - no sig effects of CO.  Placental mRNA: E.coli increase expression of IL-1b, TNF-a, IL-6, and IL-10 - CO reduces this to non-significant (p<0.05) (but no sig diff between E.coli + CO and E.coli only) |
| Sykes *et al.*, 2012 | Protein: IFNy, TNF, IL-2, IL-12, IL-1b, KCGRO, IL-5, IL-10 mRNA: IFN, TNF, IL-4, IL-10 NfKB component (p65) | Myometrium | With co-injection of LPS and Pyl A, IFN-g (p<0.05) & TNF-a (p<0.01) mRNA were sig increased, and protein IL-12, IL-1b and KC-GRO (p<0.05).  Protein IFN-g, TNFα or IL-2 not significant increase.  No sig impact of LPS or PA alone, only co-injection.  No sig increase in NFkB (p65) by LPS alone, only LPS/PA co-injection (p<0.05) |
| Domínguez Rubio *et al.*, 2014 | Uterine TNFα   Uterine iNOS and NOS levels | Uterine TNFα (ELISA) assessed 2hr after 2nd LPS injection  Uterine iNOS and NOS levels 5hr after 2nd LPS | LPS sig increase TNFα levels (p<0.05), melatonin sign reduced LPS induced TNFα (p<0.05)  NOS activity and iNOS protein levels sig higher in LPS (p<0.05), melatonin sig decreased levels (p<0.05) |
| Shynlova *et al.*, 2014 | Maternal and fetal tissues 2,6,12 hrs after LPS  IL-1b, IL-6, IL-10, TNF-a, Gm-csf/Csf2, Cxcl1, Ccl2 (plasma)  IL-1b, IL-6, IL-12, TNF-a, Csf2, Ccl2, Ccl4/Mip1b, Cxcl1 and Cxcl2/Mip2a gene expression | Plasma  Liver  Uterus  Myometrium from uterine horns  Amniotic fluid | PLASMA: at 2hr IL-1b, IL-6, IL-10, TNF-a, Gm-csf/Csf2, Cxcl1, Ccl2 sig increase in LPS (p<0.05), decreased @6hr and same as control @12hr BSCI sig (P < 0.001) attenuated TNF-a, IL-6 and Csf2 @2hr; IL-1b level non-sig (P = 0.07). LPS sig increase Ccl2 and Cxcl1, BSCI did not sig reduce.  LIVER, MYO AND DECIDUA: all chemokine genes upregulated 2hr after LPS (p<0.05-p<0.001), stayed high to 6hr. Cytokines sig increase @2hr, decrease by 6hr. BCSI sig attenuated " at 2hrs.  In liver, BCSI reduced IL-1b, IL-12, Csf2, Ccl2, Ccl4, Cxcl1 and Cxcl2 (not IL-6, TNFα or Ccl as high variability).  In myo, BCI reduced IL-1b, IL-6, IL-12, Csf2, Ccl2, Ccl4, Cxcl1 and Cxcl2 genes.  In Decidua, BCSI reduced IL-1b, IL-12, Csf2 and Ccl4 genes. (p<0.05-P<0.001)  AMNIOTIC FLUID: affected less by infection - LPS only upregulated IL-6, Cxcl1/2 (not IL-1B, TNFα and Csf2) . BCSI did not effect.  PLACENTA: LPS upregulated all cytokine and chemokine mRNA levels but models compared to maternal tissues (4x vs 42x) BUT were still upregulated 12hrs post LPS. BCSI sig reduced Cxcl2 gene expression (prompt neutrophil influx)  IL-10- in maternal plasma increase 96x, but not change in AF/placenta, slight change in liver. BCSI not affect IL-10 protein levels (but did down regulate mRNA in myo) Macrophage: BCSI not sig impact levels Neutrophils: BCSI pre-treatment inhibit LPS induced neutrophil accumulation in myometrium (p<0.01) (not decidua) |
| Paper | Maternal inflammatory markers measured | Which tissue examined | Significant change in inflammatory marker? |
| Yang *et al.*, 2014 | PTB studies showed most mice del 10-15hrs post LPS therefore killed extra group 8hrs post PS to determine inflammation: cytokine assays and Chemokine | Maternal plasma, myometrium, amniotic fluid, placenta | Pre-treatment w GR1 sig attenuated LPS induced increase in IL-1b, IL-6 & IL-12p40, TNFα, and chemokines CCL4/5 in the plasma (p<0.05) and IL-6, IL-12p70, IL-13, and IL-17 and TNFα in the myometrium (P < .05) Other cytokines (incl. IL-10) remained elevated In AF: GR1 sig attenuated LPS induced IL-6, TNFα and CCL3/4 increase (p<0.05) In placenta: GR1 attenuated LPS induced IL-6 and IL-12p70 (p<0.05)  Chemokines: Plasma: CCL4 increase sig attenuated (p<0.001) and CCL5 (p<0.01) Myo: CSF2 increase sig attenuated (p<0.05)  Placenta - effect of GR-1 AF: CCL4 increase sig attenuated (p<0.05) and CCL3 (p<0.001) |
| Filipovich *et al.*, 2015 | Total WBC count Leukocyte count Neutrophil function (Myeloperoxidase and elastase levels in uterus and placenta) Histological analysis rtPCR for: IL-1B, CCL5, TNF | Subgroup euthanised 8hr post E.coli: Blood taken Gestational tissues (uteri, fetal membrane, fetus and placenta) harvested | No sig diff in PBS or control serum.  Anti-PMN sig reduce total WBC in maternal serum at surgery (p=0.001) and 24hr post (p=0.002), and % neutrophils (p<0.001) in E.coli model (not sig 48hr after).  Anti-PMN eliminated E.coli-induced increase in circulating leukocytes, and proportion of neutrophils in uterine stroma and serum. No diff in placenta, fetal membrane or fetus E.coli sig increase myeloperoxidase in uterus (p<0.0001) - prevented by a-PMN, E.coli sig reduce myeloperoxidase in placenta (p<0.007) - prevented by an-PMN E.coli sig increase elastase in uterus (p=0.0002) and placenta (p<0.0001) - prevented by a-PMN  Anti-PMN gives no diff in cytokine gene expression (IL-1B, TNF, CCL5, PTGS2, CJA1) in placenta or uterus (IL-1B, TNF, CCL5, PTGS2 increased by E.coli p<0.05) |
| Chin *et al*., 2016 | For E.coli model: IL-1a, IL-1b, IL-6, TNF, IL-10 | Placenta & fetal membranes  Maternal decidua and myometrium | Placenta & fetal membranes: E. coli = 9.1/2,1x IL-1a, 62/6.3x IL-1b, 33/3.3x IL-6, 131/3.7x TNF, 95/4.3x IL-10 increase vs PBS. E.coli sig increase all in placenta and fetal membrane (p<0.05)  (+ )-naloxone sig reduced expression in fetal membrane (p<0.05) of IL-1b, IL-6, Tnf and IL-10, reduced by 78%, 84%, 66% and 73% respectively (trend in placenta but not sig).  Maternal decidua and myometrium (further away from injection site): E.coli sig increase all (p<0.05), (+ )-Naloxone with E. coli reduced decidual expression of IL-1a by 62% and TNF by 45%, in myometrium only Tnf sig reduced a by 43% (p<0.05). (IL-1B, IL-6, IL-10, ptgs2 no effect). Most uterine activation genes (Gja1, Oxtr, Ptger4, Ptgfr and Ptgs1; data not shown) were not elevated by E.coli - probs because of short 4hr time frame |
| Liu *et al*., 2016 | Leukocyte infiltration in decidua (density of CD44_5 cells)  L-1β, IL-6, TNF-α, CCL2 and CXCL15 in mouse uterine tissue and maternal serum  ERK1/2 and p65 in myometrium | Myometrium, placenta and blood (1h, 2h, 4h, 5h post-injection and time of LPS mice delivering 1st pup(~8hr) | LPS = increased leukocyte infiltration - 10mg NaHS sig reverse this  Mat circulation: 10mgNaHS sig reduce IL-6 (p<0.05), TNFα (p<0.001), CCL2 (p<0.05) and CXCL15 (p<0.05) vs LPS as increased by LPS (sig reduces IL-1B in negative model control, but LPS not sig increase it)  Myometrium: sig reduces L-1β, IL-6, TNF-α, CCL2 and CXCL15 mRNA expression as increased by LPS (p<0.05)  Placenta: sig reduces IL-6 and TNFα as increased by LPS (p<0.05; not IL-1B or CXCL15, and CCL2 not sig increase by LPS or reduction). NaHS alone reduced IL-1b (p<0.01) tnf (p<0.05) Ccl2 (p<0.05) vs PBS  NaHS sig attenuates LPS induced activation of ERK1/2 @ 2/4/5h post LPS (p<0.05) (not 1hr) and p65 @1/2hrs (p<0.05);quicker reaction |
| Lei *et al*., 2017 | IL-6, TNF-α and IL-10 Placental CD3+ and CD8+ T-cell infiltration | Placenta - 6hr post-surgery | No impact on mRNA levels of IL-6 and TNFα but DNAC sig increased IL-10 (vs LPS only and LPS+NAC100; p < 0.01 and p < 0.05)  DNAC sig reduced LPS-induced infiltration of CD3+Tcells and 8+ T-cells (p<0.01; not CD4/45) |
| Madaan *et al.*, 2017 | IL-1b, IL-4, IL-6, Pghs2, Ccl2, Mmp9 mRNA expression | Uteri - at time of labour collected | In negative control: LV.shGPR81 = sig higher IL-1b, IL-6, Pghs2, Ccl2, Mmp9 than sham (not IL-4) (p<0.001-0.05) In LPS model: LV.shGPR81 = sig increase IL-1b, IL-6, Pghs2, Ccl2, Mmp9 than sham (not IL-4) (p<0.001-0.05)  3,5-DHBA: sig reduced increase in IL-6 and Ccl2 mRNA expression (p<0.01 and 0.001) (IL-4 or Mmp9 not measured for these mice) |
| Paper | Maternal inflammatory markers measured | Which tissue examined | Significant change in inflammatory marker? |
| Rinaldi *et al.*, 2015 | IL-10, TNFα and IL-1B, Il-6, Cxcl1, Cxcl2 and Cxcl5 | Uterus, placenta and fetal membranes - 6hr post surgery | Does not attenuate LPS-induced expression of proinflammatory markers |
| Arenas-Hernandez *et al.*, 2019 | Casp11, Ccl22, Icam1, Ctla4, Nod1, Ccl5, IL-33, IL-6, IL-12b, IL-1a, Pycard, IL-4 | Decidual and cervix tissues | Decidua: P4 sig reduces anti-CD3e induced increase in Ccl22 (p<0.01), Icam1m (0.007) Ctka4 (0.04), Nod1 (0.02), Ccl5 (0.02). P4 also sig reduces Casp1 (p=0.007) in anti-CD3e mice despite it not sig increasing vs vehicle in anti-CD3e only mice  Cervix: P4 sig reduces anti-CD3e induced increase in IL-6 (0.004), IL-12b (0.01), IL-1a (0.007), Pycard (0.01), IL-4 (0.04) mRNA expression (p<0.05). P4 also sig reduces IL-33 (0.007) in anti-CD3e mice despite it not sig increasing vs vehicle in anti-CD3e only mice.  Myometrium: P4 only reduce anti-CD3 induced increase in IL-33 |
| Boyle *et al.*, 2019 | IL-6 protein (serum, AF)  IL-6, IL-10, Cxcl1, Ccl2 mRNA | Serum, AF  Uterus - 6h after LPS/PBS | LPS sig increased IL-6 secretion in maternal serum (p<0.0001), which was reversed by simvastatin 40ug (p<0.05) (no increase/decrease in amniotic fluid however).   Relative expression in maternal serum: LPS increased IL-6 (p<0.001), IL-10 (p<0.05), Cxcl1 (p<0.001), Ccl2 expression (p<0.001) in uterus - all sig reversed by simvastatin 40ug (p<0.01-0.05). Simvastatin 20ug had no effect on any inflammatory markers. |
| Herbert *et al.*, 2019 | LPS responsive genes Myometrial expression of: oxytocin R (Otr), Cox2, IL-1B, TNFα, IL-6 Myometrial protein levels: IL-1B, IL-6, TNFα, IFNg, CCL2/6, CXCL1/2, IL-4, IL-10   P4 responsive genes: IL-24, CXCL2, IL-11, IRAK3: P4 recues IL-11 mRNA (not IL-24, Cxcl2 or IRAK3)(p<0.001) Am & AM+P4 reduced IL-11 (p<0.01). Only AM only reduced IRAK3 (p<0.05). | tissue from left uterine horn 7h post LPS | Expression LPS increase: Cox2, IL-1B, TNFα and IL-6 (not OTR). Am/P4 individually (p<0.05) and in combo (p<0.01) only sig reduced IL-6 expression. Protein levels: P4 sig reduce IL-6 levels, and P4 & P4/Am combo sig reduce CCL1 levels (p<0.01) - none other significant. |
| Nadeau-Vallée *et al*., 2017 | mRNA transcripts: IL-1a, IL-1b, IL-6, TNFα, IL-10 and IL-12b | Uterus, decidua, placenta | LPS sig increased activation of all. Uterus: LPS sig increase IL-1b, IL-6, TNFα, IL-10, IL-12b (p<0.001-0.05; not IL-1a) - 101.1 sig reduced expression (vs LPS only) in IL-1a, IL-1b, IL-6, TNFα, IL-10 (p<0.001-0.05; not IL-12b) Decidua: LPS sig increase all (p<0.001-0.05) - 101.1 sig reduced expression of all (p<0.001-0.05) Placenta: LPS sig increase all (p<0.001-0.05) - 101.1 sig reduced expression of all (p<0.001-0.05; not IL-12b) 101.0 decreased activation of genes (apart from uterine placental IL-12b, where p=0.11) |
| Schander *et al*., 2020 | TLR4, CD14 (uterine tissues)  Neutrophil infiltration (Cervix)  Ccl5,Ccl3, Cxcl10 and Ccl2 mRNA expression | Uterus (TLR4)  Cervix (TLR4, neutrophils, chemokines)  Protein analysis 5hr post 2nd LPS | TLR4 and CD14 sig reduced in EE in uterus (0<0.05; trend in cervix but not sig)  Neutrophil infiltration sig lower in EE v CE  No sig. diff in Ccl5,Ccl3, Cxcl10 and Ccl2 mRNA expression |
| Paper | Maternal inflammatory markers measured | Which tissue examined | Significant change in inflammatory marker? |
| Wahid *et al.*, 2020 | IL-1b, IL-6, IL-12b, TNF, IL-10 mRNA expression  TLR4 and Ptafr expression | GD18.5 uterus removed - decidua and myometrium  NB vehicle control for nx missing in PBS group Placenta and fetal membranes | Decidua: cPAF sig increase IL-1b, IL-6, TNF, IL-10 mRNA expression (p<0.05; not IL-12b) - nx sig reduce IL-1b, IL-6, IL-10 mRNA expression (not TNF or IL-12b). Myometrium: cPAF sig increase IL-1b, IL-6, IL-12b, IL-10 mRNA expression (p<0.05, not TNF) - nx sig reduce IL-6, IL-12b,IL-10 mRNA expression (p<0.05, not IL-1B or TNF) Placenta: cPAF sig increase IL-1b, IL-6 mRNA expression (p<0.05, not IL-12b, IL-10, TNF) - +nx sig reduce both (p<0.05). Fetal mem: cPAF only sig increase IL-10 (p<0.05) - +nx not sig reduce  Tlr4 expression not sig altered by cPAF in any tissue. Ptafr expression sig reduced only in fetal membranes (p<0.05), not sig changed by nx Ptgs2 sig increased by CPAG in decidua and myometrium; only sig reduced by nx in decidua. |
| Chen *et al*., 2012 | 1 LPS injection Placental mRNA expression & Maternal serum proinflammatory cytokine levels: TNFα, IL-1B, IL-6, IL-8  Placental mRNA & maternal serum anti-inflammatory cytokines: IL-4, IL-10 | Placenta and maternal serum | Proinflammatory mRNA: LPS sig increase all (P<0.01) and Zn sig prevent increase for all (IL-1B & IL-6 p<0.01, TNFα and IL-8 p<0.05) Protein: LPS sig increase (p<0.01) and Zn sig prevent increase for all (p<0.01) Anti-inflammatory mRNA: LPS sig reduce IL-4 (p<0.05), expression sig increased by Zn + LPS (p<0.01) whereas LPS sig increase IL-10 (p<0.05), expression sig reduced by Zn + LPS (p<0.01) Protein: LPS sig reduce IL-4 (p<0.05), levels sig increased by Zn + LPS (p<0.01) whereas for IL-10, Zn sig increase for control and LPS group vs no-Zn (p<0.01) (but LPS no effect on own)  NFkB activation observed in LPS group but not with Zn treatment |
| Toda *et al.*, 2016 | IL-6 - serum and uterine  KC, keratinocyte-derived protein chemokine; MCP-1, monocyte chemoattractant protein-1; MCP-5, monocyte chemoattractant protein-5 - uterine total RNA expression | Blood taken just before LPS and 1hr after 2nd LPS  Uterine tissues taken 1hr after LPS | IMD sig reduce serum protein levels and uterine mRNA expression of IL-6 (p<0.05)  RNA expression of Kc, MCp-1 and Mip-2 sig reduced by IMD (p<0.05) |
| Agrawal *et al*., 2018 | total RNA: IL-1β, TNFα,IL-6, iNOS, Nlrp3, CCL5, CCL2/MCP-1, IL-22, IL-22 receptor), IL-10, LC3B, and Gapdh   E.coli colony count in tissue/AF   Cytokine: TNF, IL-6, and IFN-γ, MIP-1β, and anti-inflammatory IL-10   M1&M2 Macrophage analysis | Tissue harvest mice protocol: 1. Sterile IU with IV dilutant simultaneous 2. E.coli IU with IV dilutant simultaneous 3. Sterile IU with IV SP-A simultaneous 4. E.coli IU with IVSPA simultaneous 8h post = euthanised and uterus, decisualised endometrium caps and placenta removed. amniotic fluid removed | E.coli upregulated IL-1β, TNF, IL-6, iNOS, NLRP3, CCL2/MCP-1, and IL-22 (decidua only) but not CCL5, IL-10 or IL-22-R - SPA tended to counteract changes, not sig  No inhibition of bacterial growth in decidual caps or AF (no sig diff between any group)  E.coli sig increase TNF, IL-6, and IFN-γ, MIP-1β levels (p<0.01), and SP-A sig reduce levels (p<0.05) No sig effect of E.coli/SP-A on anti-inflammatory IL-10  E.coli polarises decidual macrophages to M1 (CD11C) phenotype (decrease in M2(CD206) & CD11c+ and CD206+) - polarise reversed with SP-A |
| Paper | Maternal inflammatory markers measured | Which tissue examined | Significant change in inflammatory marker? |
| Fu *et al.*, 2019 | Placental Tnfα, IL-1β, Mcp1 and Mip2 mRNAs  Placental p-IκBα, IκBα, NF-κB p65 and NF-κB p50 (Western blotting)  Placental NF-κB p65 positive nucleus | Placenta | LPS sig increase placental Tnfα, IL-1β, Mcp1 and Mip2 mRNAs (p<0.01), VD3 sig reduce all (p<0.01)  LPS sig increase p-IκBα, IκBα, NF-κB p65 and NF-κB p50 (p<0.01), VD3 sig reduce all (p-IκBα& IκBα: p<0.01, NF-κB p65 and NF-κB p50: 0<0.01)  p65+ nucleus: LPS sig increase % (p<0.01), VD3 sig reduce (p<0.05) |
| Zhang *et al.*, 2020 | IL-1B, IL-6, TNFα in serum and AF (ELISA)  Leukocyte infiltration  Uterus NFkB activation and iNOS protein level | 12hr post 1st LPS/NS subset killed and uterus (western blot), placenta (H&E and immunohistochemistry), AF and blood collected (ELISA) | EPO sig reduced LPS induced increase in serum and AF IL-1B (P < 0.0001 S/ < 0.001 AF), IL-6 (P < 0.001 S/ < 0.0001 AF) and TNFα (P < 0.0001 S/ < 0.001 AF) (LPS sig increase all P < 0.0001)  LPS sig increase leukocyte infiltration to placenta, EPO prevented this infiltration.  EPO sig reduced LPS induced increase in uterine NFkB activation (p65 phosphorylation; p<0.01) and iNOS protein level (p<0.05) |

| 1. Effect of the intervention on other maternal/fetal outcomes | | |
| --- | --- | --- |
| Paper | Other maternal/fetal health outcomes measured | Results of other maternal/fetal outcomes |
| Schmitz *et al.*, 2007 | Cervical ripening - looking at epithelial gland secretory vacuoles and trichrome staining for order of collagen fibres | LPS only = glandular epithelium has increased volume of epithelial glands with secretory vacuoles. Trichome staining shows disordered collagen fibres.  LPS + rolipram = noncompliant tissue; denser collagen fibres (suggesting undeveloped glands) and absence of secretory vacuoles. |
| Peltier *et al*., 2013 | Pup weight @ PN day 1 | PW sig reduced by E.coli, sig improved by CO treatment in E.coli model (P=0.001) |
| Sykes *et al.*, 2012 | Uterine contractility - uteri mounted on myograph   Pup brain inflammation - NfKB and COX2  Myo COX2 | Pyl A inhibited myometrial contractility from a concentration of 10 lm (P < 0.1), with complete inhibition seen with 100 lm (P < 0.001)  Sig reduction in p65 with LPS only, but no sig change with PA. No sig diff in pup brain COX2.  No sig. increase of myo COX2 by LPS alone, only LPS/PA co-injection (p<0.05) |
| Domínguez Rubio *et al.*, 2014 | Uterine PG levels and COX expression 5hr after 2nd LPS injection  Representative photos of fetus from GD15 5hr after 2nd LPS/vehicle injection  The body weight of pups was recorded at day 1, 8, 15, and 22. Representative photos of pups day 8  Cervix morphology The time of eye-opening, hair growth, and nail and tooth eruption | Melatonin significantly prevented the increase in uterine PGE2 & PGF2a and COX2 levels induced by LPS (p<0.05 for increase and reduction)  Decrease in cerebral irrigation induced by LPS, which was not observed in LPS & melatonin  No impact of LPS or melatonin on pup weight  All pups have similar appearance  LPS caused vaginal bleeding and less defined interstices - which is not seen in mice who had melatonin  No difference in the time of eye-opening, hair growth, and nail and tooth eruption |
| Shynlova *et al.*, 2014 | Foetal and placental weight | Fetal weight of term BCSI pups = sig higher than term BCSI&LPS pups Term birth weight: LPS - NA (none to term) LPS + BCSI - 0.97 ± 0.15 * (p<0.005 vs BCSI only) BCSI - 1.3 ± 0.12 Vehicle - 1.1 ± 0.17  Term placental weight:  LPS - NA (none to term) LPS + BCSI - 0.098 ± 0.007 BCSI - 0.094 ± 0.015 Vehicle - 0.097 ± 0.018 (no sig diff) |
| Paper | Other maternal/fetal health outcomes measured | Results of other maternal/fetal outcomes |
| Yang *et al.*, 2014 | Maternal plasma progesterone  Fetal sex ratio, weight and size | LPS sig reduced P4: - control: 68 ± 4.6 ng/ml - LPS: 42 ± 7.4 (p<0.05) - LPS + GR1: 38 ± 4.5 (no sig diff from LPS) - GR1 only: 59.1 ± 1.7 - comparable to control (p>0.05)  No effect of sex on PTB  No effect of GR-1 on average fetal weight or litter size for term pups in any group. |
| Filipovich *et al.*, 2015 | Uterine and placental COX2, CJA1 | Gap junction alpha-1 protein, also known as connexin 43 - no effect of E.coli or anti-PMN  E.coli increase COX2, but no change with PMN |
| Chin *et al*., 2016 | FGR   COX (ptsg2) in myo and decidua | In the 45% (9/20) LPS-treated dams remaining undelivered at g.d. 18.5, higher incidence of severe FGR and fetal death (18.9%, 10/53) compared to PBS-treated controls (1/115, 0.8%, p = 0.023). Not sig reversed by naloxone.  Naloxone also not affect birth weight in E,coli  COX: increased by E.coli, but naloxone did not sig reduce (trend) |
| Liu *et al*., 2016 | N/A | N/A |
| Lei *et al*., 2017 | Pup weight at PND5  Neurobehavioral at PND 5 and 9   Microglial activation at PND 17 | No sig diff in pup weight between PBS or LPS  DNAC and NAC sig improved negative geotaxis or cliff aversion tests at PND5&9 (LPS have impaired performance) (p<0.001-0.05 for different tasks)  Microglia: less LPS induced microglial activation in DNAC (LPS/NAC) p < 0.001 |
| Madaan *et al.*, 2017 | Pghs2 Gpr81 mRNA in uterus | DHBA reduced LPS induced Pghs2 and Gpr81 expression p<0.001 and p<0.05 |
| Rinaldi *et al.*, 2015 | Ptgs2 and 15-Hpgd (key enzymes responsible for regulating prostaglandin synthesis and breakdown) | In uterus: EA4 sig reduced LPS induced increased in 15Hpgd expression (p<0.001), whereas sig increase Ptgs2 (p<0.05) In placenta: EA4 sig increased Ptgs2 levels (p<0.001) in LPS model, no effect on 15Hpgd In fetal membranes, ELA4 only sig increase Ptgs2 in LPS model (p<0.05) (no effect on 15Hpgd)  No effect on cytokines TNFα, IL-1B. |
| Arenas-Hernandez *et al.*, 2019 | Pup size | Pups from all groups similar size |
| Boyle *et al.*, 2019 | Cx43, Cox-2 mRNA in uterus | LPS had no effect on IL-10, Cox2 or Cx43. Simvastatin had no effect on IL-10 or Cox2, but did sig reduce Cx43 in LPS model. |
| Herbert *et al.*, 2019 | Ex vivo myometrial contractility | Ex vivo myometrial contractility was not altered by Am and P4 |
| Nadeau-Vallée *et al*., 2017 | New-born weight  Fetal brain inflammation | No sig difference in new-born weight in any of the groups  LPS sig increase IL-1a, IL-1b, IL-6, TNFα, IL-12b (p<0.001-0.05; not IL-10) - 101.1 sig reduced expression (vs LPS only) of all (p<0.001-0.05) |
| Paper | Other maternal/fetal health outcomes measured | Results of other maternal/fetal outcomes |
| Schander *et al*., 2020 | Serum cortisone  Cervical MMP  COX2, PGF2a, PGE2, iNOS and NOS activity | LPS sig increase [cortisone] in CE, EE sig prevents this increase (P<0.05)   EE sig prevents cervical MMP9 gelatinase activity (p<0.05) (no effect of LPS on MMP2). Mmp8 mRNA expression in cervix: no change with EE  EE sig prevents increase in uterine NOS activity (p<0.05) (not iNOS, PGE2, PGF2 or COX2) EE sig prevents increase in cervical PGE2 synthesis and NOS activity (p<0.05) (not iNOS or COX2) |
| Wahid *et al.*, 2020 | Fetal placenta weight at GD18.5  Pups weighed 12-24h post-delivery and PND8 and 21  Ptgs2 expression in the decidua and myometrium | I.P.: placental weight and fetal-placental weight no diff between any group +nx: fetal weight 6% smaller in cPAF+nx vs +nx-only (p=0.024); nx not prevent reduced fetal weight  I.U.: no diff in placental weight, but nx prevents cPAF reduction in fetal: placental weight (p<0.05) +nx did not improve fetal weight  cPAF sig. increase Ptgs2 expression in the decidua and myometrium (p<0.05), +nx sig reduce in decidua (p<0.05, not myo) - I.P. injection |
| Chen *et al*., 2012 | Placental weight  Of live fetuses:  fetal weight crown-rump length  COX2 placental expression  MT1/2 mRNA expression in placenta and liver (melatonin receptor subunits)  Skeletal retardation | Placental weight control: 0.106 ± 0.006 Zn: 0.102 ± 0.003 LPS: 0.085 ± 0.002** (P<0.01 vs control/zn) LPS + Zn: 0.103 ± 0.003 ** (P<0.01 vs LPS)  Fetal weight: control: 1.42 ± 0.030  Zn: 1.41 ± 0.018 LPS: 1.16 ± 0.029** (P<0.01 vs control/zn) LPS + Zn: 1.32 ± 0.035 ** (P<0.01 vs LPS)  Crown-rump length: control: 2.56 ± 0.018 ±  Zn: 2.57 ± 0.023 LPS: 2.36 ± 0.031** (P<0.01 vs control/zn) LPS + Zn: 2.50 ± 0.020** (P<0.01 vs LPS)  COX2: LPS sig increase expression (p<0.01), prevented in Zn + LPS (p<0.05)  MT expression Liver: LPS sig increase MT1&2 expression (p<0.01), no effect of Zn Placenta: No effect of LPS or Zn on MT1/2  Skeletal: LPS mice had fewer ossification centres in caudal vertebrae and fetal supraoccipital - Zn sig attenuated skeletal development retardation (p<0.05) |
| Toda *et al.*, 2016 | N/A | N/A |
| Agrawal *et al*., 2018 | N/A | N/A |
| Fu *et al.*, 2019 | Maternal serum calcium and phosphorus  Maternal serum 25(OH)D levels and placental Cyp27b1mRNA and Cyp24a1 mRNA  Placental VDR immunohistochemistry  Serum estrogen and progesterone  mRNA of placental genes for P4 biosynthesis (star, cyp11a1, 3bHsd) and levels of enzymes in placenta  Maternal ERa & b in mouse placenta, and Cyp19 (enzyme for E2 synth)  Serum PGF2  Placental COX2 | No sig diff in calcium or phosphorus  VD sig increase 25(OH)D levels and placental Cyp24a1 mRNA expression in both control (p<0.01) and LPS (p<0.05) (not placental Cyp27b1 mRNA) - no effect of LPS  VDR: VD3 sig increase in control and LPS (p<0.01) - no effect of LPS  LPS sig increase e2 (p<0.05) and P4 (p<0.01) - only P4 levels restored by VD3 (p<0.05)  LPS sig reduce levels and expression of cyp11a1 and 3bHsd, and VD3 sig restores levels (p<0.05 for all) (no effect on star)  LPS sig increase CYP19, ERa and ERb (p<0.01), VD3 sig reduces CYp19 and ERb (p<0.05; not ERa)  LPS sig increase PGF2 (p<0.01), VD3 sig reduce (p<0.01)  LPS sig increase relative Cox2 mRNA (p<0.05), COX2/a-tubulin (p<0.01) and COX2-+ve cells (p<0.01), VD3 sig restore all (Cox2 p<0.05, others p<0.01) |
| Zhang *et al.*, 2020 | Placental PGE2 in placental trophoblast cells  Placental PD-L1 expression | EPO on own no effect on PGE2 production, but sig attenuated LPS-induced upregulation of PGE2  EPO increased LPS induced reduction in placental PD-L1 expression |
